# Results of a feasibility study of the FReSH START intervention to improve quality of life and other outcomes in people who repeatedly self-harm (Function REplacement in repeated Self-Harm: Standardising Therapeutic Assessment and the Related Therapy)

**DOI:** 10.1186/s40814-025-01644-2

**Published:** 2025-05-15

**Authors:** Bethan Copsey, Alexandra Wright-Hughes, Amanda Farrin, Cara Gates, Kate Farley, Cathy Brennan, Dean McMillan, Christopher D. Graham, Chris Bojke, Richard Mattock, Adam Martin, Petra Bijsterveld, Judith Horrocks, Suzanne Hartley, Marsha McAdam, Dafydd Hammond-Jones, Louise D. Bryant, Allan House, Elspeth Guthrie, the FReSH START lived experience group

**Affiliations:** 1 Clinical Trials Research Unit, Leeds Institute of Clinical Trials Research, University of Leeds, Leeds, UK; 2 Leeds Institute of Health Sciences, University of Leeds, Leeds, UK; 3 Department of Health Sciences, University of York, York, UK; 4 Department of Psychological Sciences & Health, University of Strathclyde, Glasgow, UK; 5 Academic Unit of Health Economics, Leeds Institute of Health Sciences, University of Leeds, Leeds, UK; 6 Patient advocate, Expert by experience, Manchester, UK; 7 Leeds Teaching Hospitals NHS Trust, Leeds, UK

**Keywords:** Self-harm, Mental health, Feasibility study, Therapy, CBT, ACT, PIT

## Abstract

**Background:**

Self-harm is a major public health challenge with estimated lifetime prevalence of 5–6% and 220,000 hospital attendances annually in England and Wales. Repetition of self-harm is common with 70% of hospital attenders reporting previous self-harm. Multiple repetition bears a significant cost to individuals and healthcare systems. A recent Cochrane review showed little evidence for the benefit of existing psychological therapies for people who repeatedly self-harm. Considering multiple possible functions of self-harm, we modified three existing psychological therapies for use with people who self-harm multiple times. To inform the design of a definitive multi-centre randomised controlled trial (RCT) and assess the feasibility of an RCT, this mixed-methods feasibility study assessed intervention delivery and acceptability.

**Methods:**

A single arm (comprising three modalities), non-controlled, multi-centre feasibility trial aimed to recruit 30 participants aged 16 years or older and reporting both recent and recurring self-harm episodes. The FReSH START intervention included 12 individual sessions over a maximum 6 months comprising one of three psychological therapies, each modified specifically for use with people who have self-harmed multiple times: Cognitive Behavioural Therapy, Acceptance and Commitment Therapy, and Psychodynamic Interpersonal Therapy. Follow-up was via participant reported outcomes using postal questionnaires at 6 months and monthly text messages.

A parallel qualitative study interviewed a sample of therapists and participants to refine the intervention and logic model ahead of a definitive RCT.

**Results:**

We reached our target of 30 recruited participants and 15 therapists delivered the intervention in a way that was acceptable to participants. However, follow-up rates for the 6-month questionnaire were lower than expected at 53.3% (*n* = 16/30). To improve follow-up, in the definitive RCT, we plan to use online questionnaires, provide vouchers and behaviourally-informed letters to incentivise questionnaire return, and include follow-up via routinely collected data. Intervention fidelity also requires some improvement in specific areas; thus we plan to amend the intervention therapist training accordingly.

**Conclusions:**

Despite disruption due to the COVID-19 pandemic, we conclude that delivery of a definitive trial of adapted psychological therapies for people who repeatedly self-harm is feasible with modifications to study processes to improve intervention fidelity and participant retention.

Trial registration.

ISRCTN16049211.

**Supplementary Information:**

The online version contains supplementary material available at 10.1186/s40814-025-01644-2.

## Key messages regarding feasibility

- What uncertainties existed regarding the feasibility?

The main uncertainties regarding the therapies were whether mental health professionals could be trained and successfully deliver the therapies as intended (incorporating the elements from the original therapy as well as the FReSH START components) and whether participants would attend treatment sessions. In terms of the trial, the main uncertainties were whether participants could be recruited to the study, and successfully followed-up 6 months after study enrollment.

- What are the key feasibility findings?

During times of difficulty due to pressure from COVID-19, we were able to train therapists and recruit participants into a trial for an intervention to improve the quality of life for people who self-harm. We delivered the intervention with high levels of attendance at therapy sessions. However, we encountered challenges to achieving treatment fidelity in some cases and in obtaining participant follow-up data.

- What are the implications of the feasibility findings for the design of the main study?

To improve follow-up rates, we plan to offer online questionnaire completion, include a letter from our lived experience group, and provide a voucher for questionnaire completion. We originally planned to follow-up participants at 6 and 12 months; however, we will now also include short questionnaires at 3 and 9 months to reduce the recall period for self-reported health economics data. We will also supplement questionnaire data with routinely collected data on self-harm hospital attendance via Hospital Episode Statistics.

To improve intervention fidelity, intervention training will be amended to focus on elements with poor fidelity in the feasibility study, and incorporate additional therapist readiness checks. Fidelity will be assessed frequently for the first set of participants seen by each therapist. We will also implement an additional clinical check prior to recruitment to ensure that participants are suitable for therapy.

An internal pilot will be used in the definitive trial to assess the changes in intervention training and follow-up processes.

## Background

Self-harm is a major public health challenge with estimated lifetime prevalence of 5–6% [1–3] and 220,000 hospital attendances annually in England and Wales [4, 5]. Repetition of self-harm is common with 70% of hospital attenders reporting previous episodes of self-harm [6]. Up to 20% of those who present to hospital report a history of over five acts and about 25% attend hospital for a subsequent act during 18-month follow-up [6]. For those seen in hospital after at least their third attendance for self-harm, more than 50% will go on to a further attendance following self-harm [6]. We know most about hospital attendance because of ease of data collection, but it is apparent that many additional episodes do not lead to hospital attendance [7, 8]. It is therefore estimated that some 40–50,000 hospital attendances a year are accounted for by those who repeatedly self-harm, with as many episodes again not leading to hospital attendance [9].

Repeated self-harm especially is associated with other problems such as depression; misuse of alcohol; poor quality of life and problems with interpersonal and social functioning [10]. Repetition of self-harm compared with single episode self-harm is associated with an increased risk of suicide [11]. Multiple repetition bears a significant cost to the individual, the healthcare system and the economy as a whole. Total healthcare costs rise significantly in the 6-month period following hospital attendance for fifth (or greater) episodes compared to a first episode [12].

Existing literature provides a useful distinction between acts of self-harm that are a response to recent stressors associated with acute distress and often not repeated once the stress is resolved, and multiple (repeated) acts associated with longer-term social and psychological problems [13]. A recent Cochrane review showed little evidence for the benefit of existing psychological therapies to treat repeated self-harm which is not underpinned by recent environmental stressors [14]. Therapies that have been studied are primarily cognitive behavioural therapy (CBT)-based psychotherapy but also include dialectical behaviour therapy (DBT) and Psychodynamic psychotherapy [14]. The therapies that have been studied are usually intensive, of long duration (6–12 months), in specialist services, are highly selective and require specialist therapists; and there is no published evidence of their cost-effectiveness.

Existing psychological therapies, which have shown limited efficacy, have focused on reducing self-harm directly. Despite the importance of reducing repetition, based on clinical experience, we considered that a therapeutic approach working with service users to identify valued (positive) goals could be a more acceptable approach than therapies focused on reducing self-harm. A Q-sort study was conducted to identify functions for self-harm [15]. Following this, three existing psychological therapies were adapted to focus on values and life goals as illustrated by these functions, with the aim of identifying ways of achieving this goals. The modified therapies include cognitive behavioural therapy (CBT), acceptance and commitment therapy (ACT) and psychodynamic interpersonal therapy (PIT), which are all delivered in NHS practice and can be delivered by mental health professionals with brief initial training. Although the therapies retain their own essential components, all three incorporate the same specific adaptation in order to provide a new approach to self-harm, by attempting to elucidate and focus on positive goals and values. This contrasts with previous interventions, which emphasise tackling negative drivers such as affect dysregulation or hopelessness [15, 16].

This feasibility study examined an approach to modifying existing therapies specifically for use with people who repeatedly self-harm and aimed to:

1. Assess intervention delivery and acceptability
2. Assess the feasibility of conducting a definitive randomised controlled trial (RCT) of the FReSH START Intervention versus Usual Care (UC) for adults, and
3. Inform the design of the definitive RCT

## Methods

### Design

A single arm, non-controlled, feasibility trial of the FReSH START intervention, took place across three UK sites, which aimed to recruit 30 participants aged 16 years or older who repeatedly self-harm.

The FReSH START intervention comprised three psychological therapies, each modified specifically for use with people who repeatedly self-harm and designed to be readily learned by mental health staff and delivered in 12 sessions or fewer over a maximum 6 months. Follow-up was via participant reported outcomes using postal questionnaires at 6 months and monthly text message.

A parallel qualitative study interviewed a sample of therapists and participants to refine the intervention and logic model ahead of the definitive RCT.

### Participants

To ensure that our intervention is compatible with NHS practice, we recruited through mechanisms which mirror NHS pathways. We recruited people who presented to hospital Emergency Departments (ED) with self-harm. We planned to recruit through adult mental health teams and primary care as well as ED, but we found this was not feasible as it was not possible to ensure compliance to NICE guidelines for self-harm using these routes [17]. Inclusion criteria were assessed by the clinical team in the ED during the person’s psychosocial assessment. Exclusion criteria were assessed by the study researcher if the person consented to researcher contact.

#### Inclusion criteria

- Aged 16 years or over,
- Presenting at ED, adult mental health services or general practice as a consequence of self-harm within the last 8 weeks, defined as: intentional acts, regardless of reported suicidal intent, that directly harm a person’s own body. This includes methods like cutting, burning, scratching, banging or hitting parts of the body or interfering with wound healing, and self-poisoning, such as taking overdoses of drugs,
- Has mental capacity to provide fully informed written consent, and
- Self-harm episode in the preceding three months that is at least their 3rd episode in the preceding 12 months and their lifetime 4th or more episodes.

Criteria on prior self-harm episodes was based on previous research that those who have harmed themselves four or more times have a >50% chance of going on to repeat hospital attendance for self-harm [18].

#### Exclusion criteria

- Receiving a specific psychological intervention that is similar to the trial intervention, or where a specific intervention is indicated for a related condition (e.g. anorexia nervosa or drug addiction) and would conflict with trial participation,
- Lacks capacity to comply with study requirements,
- Insufficient proficiency in English to contribute to the data collection,
- Known risk of violence (for example reported by ED or liaison psychiatry staff),
- 16 or 17 years of age and attending school and/or not eligible for treatment by local adult mental health services, or
- Researcher unable to contact participant within eight weeks following self-harm event.

### Intervention

All participants were allocated to the FReSH START intervention, which was delivered by a mental health professional recruited and trained in one of the modified psychological therapies: Cognitive Behavioural Therapy, Acceptance and Commitment Therapy, and Psychodynamic Interpersonal Therapy. The therapies were each adapted to focus on self-harm and incorporated a functional assessment of self-harm in the initial sessions of therapy.

The therapy was designed to be delivered by qualified mental health professionals (mental health nurses, psychologists, occupational therapists, social workers, psychiatrists or counsellors) who worked in an acute mental health setting and had prior experience of working with people who self-harm and of managing risk. Initial therapist training consisted of a face to face 3-day workshop delivered by the co-investigator therapy leads, with additional online materials provided as necessary, followed by on-going remote therapy-specific group supervision (maximum 3–4 people per group) lasting 90 min at least every 2 weeks. Clinical risk was managed by the therapists with support from their acute mental health teams.

The therapy training was disrupted by the COVID-19 pandemic and the shutdown of the study for 6 months. The 3 days of PIT and ACT training was completed. One day of CBT training was completed. After the study was re-started, PIT, ACT and CBT therapists received top up training on-line. In addition, another 3 days of online ACT training was conducted to include a further site and new therapists. Subsequently, all training was modified so it could all be delivered online with 2–3 h sessions over a 2-week period of time (PIT 4 × 2-h sessions, ACT 3 × 3-h sessions, CBT 3 × 3–4 h sessions).

Therapists were randomly allocated to participants as far as possible; however, the assigned therapist could deviate from the randomisation where necessary, for example, due to therapist availability, workload, or if the participant had a strong preference for the therapist’s gender. Participants received the therapy their assigned therapist was trained to deliver.

Recruited participants received up to 12 sessions lasting 45 to 50 min of the intervention over a maximum of 6 months, where possible on a weekly basis; with an option of 1–2 additional sessions (typically by telephone) within 3 months of completion of therapy.

The therapy was originally designed to be delivered face to face. However, as a result of the COVID-19 pandemic, this changed to either face to face (with appropriate social distancing and personal protective equipment) or remote therapy via a secure NHS video link, determined by current Trust practice, therapist and participant preference.

The adaptation of the therapy for self-harm was based upon findings from the first part of the research programme which included two systematic reviews [19, 20], a review of self-help material for self-harm available on the internet [21] and a study exploring the underlying motivations of self-harm [15]. Therapists were encouraged to adopt a supportive, non-judgemental, compassionate approach to their clients. In the first session, the therapeutic assessment focused upon elements of practice that people who self-harm find particularly helpful, in particular, recognising the positive benefits that they experience from self-harm (e.g. relief of tension, sense of control, opportunities for self-care) and the important role it plays in their lives [20] and the potential underlying drivers. Prominence was given to formulation and understanding of the potential positive or protective benefits self-harm plays, by undertaking a functional analysis of self-harm. The first session also addressed participant expectations regarding treatment and aimed to work collaboratively to develop a positive rationale for psychological treatment and goal setting. A detailed risk assessment and safety planning were also key aspects of the first session.

Subsequent sessions depending upon the type of therapy focused on the following:

- Considering ways to improve well-being via discussion of overarching values and goals;
- Helping participants notice patterns of thoughts, feelings, and how these might influence their choices they make in life and in relationships;
- Discussion of problematic relationships past and present and ways in which these can be improved;
- Practising making choices that make life better for the participant, which may involve finding different ways to engage with the situations or strong/unpleasant feelings and impulses that quickly lead to self-harm.
- CBT focused on function and cognitions; ACT on improving psychological flexibility; and PIT on feelings and interpersonal relationship problems.

Details of each of the approaches and the TIDIER checklists are provided in Additional File 1.

Sessions were audio-recorded to facilitate fidelity assessment and therapist supervision. For comparability across therapies, we developed a common fidelity measure to capture safety and FReSH START components, completed by the therapists and retrospectively by study researchers using audio recordings for all participants; this was based on the first 3 sessions. The trial therapy leads assessed therapy-specific fidelity, using audio recordings on a random sample of sessions (one session per participant where available), against a subset of items from existing scales: Sheffield Psychotherapy Rating Scale for PIT and CBT and the ACT Fidelity Measure (ACT-FM) for ACT [22–24]. Therapy-specific fidelity was based on the 4 th session onwards.

### Baseline and follow-up data collection

Participant demographics were collected at baseline, including age, gender, ethnicity, sexuality, education, employment, details of the presenting self-harm event, self-harm history, comorbidities, alcohol and substance use, current co-morbid physical and mental health.

Monthly 2-way text messages were sent for 6 months to record general health and occurrence of self-harm.

The following participant reported data were collected at baseline and via postal questionnaires at 6 months:

- Clinical Outcomes in Routine Evaluation–Outcome Measure (CORE-OM) [25] (pre-specified planned primary outcome for definitive RCT and used to construct QALYs (quality-adjusted life years) for health economic analysis)
- Hopelessness—Beck Hopelessness Scale [26]
- Depression–PHQ-9 [27]
- Social connectedness—The Social Connectedness Scale-Revised [28, 29]
- Self-reported episodes of self-harm (frequency, whether self-injury or self-poisoning or both, related hospital attendance)
- Self-reported resource use—primary and community care and medications and private financial burden due to self-harm.

### Outcomes of the feasibility study

The outcomes of the feasibility study were:

#### Recruitment methods, uptake and follow-up

The feasibility and success of the recruitment strategy was evaluated by summarising the screening, eligibility, consent and registration processes, including numbers of participants involved during each stage. Participant retention during follow-up, including number of participants completing/withdrawing from the study and reasons for withdrawal were summarised.

#### Intervention delivery, acceptability and fidelity

Intervention delivery and acceptability were assessed by summarising the proportion of participants successfully completing therapy (attending all planned therapy sessions), the number and reasons for dropout, overall and by therapy. Intervention fidelity and adherence was summarised in terms of the number of therapy sessions attended, evidence of delivery of safety and FReSHSTART intervention components (relating to the self-harm approach) and the specific therapy.

##### Follow-up data collection

The feasibility and success of obtaining self-reported self-harm data via monthly text message was assessed by summarising the proportion of participants responding during the 6-month follow-up period. Acceptability of 6-month questionnaires was assessed by summarising overall and item level completion.

##### Statistical outcomes

To check our assumptions for the sample size for the definitive trial, we estimated the variability (standard deviation) of the self-reported outcomes at baseline and 6-months, and report summary statistics and 95% confidence intervals.

##### Health economics outcomes

We piloted service usage and health related quality of life (HRQoL) questionnaires to confirm the feasibility of identifying costs and quality adjusted life years (QALYs) during the definitive trial. We also trialled methods to estimate intervention costs based on case report forms completed by therapists and supervisors.

##### Progression criteria

We had pre-specified progression criteria on the rate of recruitment, follow-up data collection, intervention fidelity of therapies and intervention acceptability for participants (Table 1).

**Table 1:** Progression criteria

	Red	Amber	Green (95% CI)
Recruitment—Progression criteria 1: Average number of participants recruited per month	< 4	4–7	> 7
Follow-up—Progression criteria 2: Proportion of registered participants completing the CORE-OM at 6 months	< 60%	60–75%	> 75% (95% CI: 59.5%, 90.5%)
Intervention delivery—Progression criteria 3: Proportion of participants where the therapist achieved intervention fidelity in terms of safety and FReSH START components	< 60%	60–80%	> 80% (95% CI: 65.7%, 94.3%)
Intervention acceptability—Progression criteria 4: Proportion of registered participants attending their 1 st treatment session	< 50%	50–70%	> 70% (95% CI: 53.6%, 86.4%)

##### Sample size

We planned to recruit 30 participants to provide sufficient data to assess progression criteria to inform the decision to move to a full-scale definitive randomised evaluation. As this study is designed to determine the feasibility of a confirmatory trial and not to assess proof of concept or evaluate effectiveness, formal power calculations are not appropriate. With 30 participants, the 95% confidence interval (CI) around our green (go) criteria overlapped the values in the red zone by less than 1%, relating to our stopping criteria. For example, the green (go) criteria for proportion of participants completing follow-up at 6 months is > 75% with a 95% confidence of 59.5% to 90.5%; the red zone is 0 to < 60%. Therefore, with 30 patients, by meeting the green (go) criteria, we could be sufficiently confident that follow-up, delivery and acceptability would not fall to unacceptable levels as defined by the red (stop) criteria.

##### Randomisation

Randomisation of therapists was performed using permuted blocks where the block size was based on the number of therapists at the corresponding site.

Participants, therapists and researchers were unblinded to the therapy being delivered, due to the nature of talking therapies.

##### Trial oversight

A programme steering committee provided overall supervision of the study, in particular, study progress, adherence to protocol, participant safety and consideration of new information.

##### Statistical analysis

Statistical analysis was primarily descriptive and used confidence interval (CI) estimates rather than hypothesis testing for data relating to outcomes and progression criteria. For categorical outcomes, the number and proportion of participants was reported. For continuous questionnaire outcomes, summary statistics and 95% confidence intervals are reported.

An investigation of the therapy and therapist clustering effect (ICC) was carried out for the CORE-OM at 6 months. The therapy ICC was calculated using a mixed effects regression model with a random effect for therapy, and adjustment for baseline CORE-OM and centre. However, the 95% confidence interval of the therapist ICC could not be calculated due to small numbers of participants per therapist.

Missing data were not imputed, other than for calculating questionnaire scores where some but not all items were missing. Details of item-level imputation are in Additional File 2.

All analyses were conducted on the intention-to-treat (ITT) population, including all participants regardless of non-compliance with the protocol or withdrawal from the study. Participants were categorised based on the therapist assigned to deliver their treatment and the corresponding therapy.

##### Health economics analysis

Costs were estimated by multiplying resource usage from the feasibility analysis questionnaires by unit costs obtained from national databases [30–32]. Cost categories included intervention costs, health and social care costs and societal costs through work absenteeism and private expenditure. Participant questionnaire data were obtained over the prior 3 months, as this was considered the longest period of time without incurring a high risk of recall bias. Consequently, costs are not obtained for the full 6 month follow up period in the feasibility study. In the definitive trial, participant questionnaires will be collected every 3 months thus covering the full follow up period. A full description of the costing methods is provided in Additional File 5.

Health-related quality of life (HRQoL) was captured using the CORE-6D, which is derived from 6 questions in the CORE-OM, relating to emotional or physical health, with three severity responses, and consequently 729 unique health states [33]. Utility scores for the health states were assigned using an algorithm developed by Mavranezouli et al. [33]. Generic measures such as the EQ-5D were not considered appropriate as they focus on physical attributes of HRQoL and may not be sensitive to mental health attributes. Quality adjusted life years were calculated across 6 months assuming a linear relationship between HRQoL at baseline and follow-up. If participants died, QALYs were calculated assuming baseline HRQoL applied up to the date of death.

##### Qualitative methods and analysis

The feasibility study included embedded qualitative interviews to explore the acceptability of the approach from a participant and therapist perspective and to gain insights into how participants felt the therapy worked for them.

During the study consent process, participants were asked to agree to being contacted again to invite them to participate in an interview. Study participation was not contingent upon consent to interview and participants could decline to be interviewed when they were invited. Consenting participants were interviewed by telephone or video call at the end of therapy to explore their experiences and the perceived impact on their social and psychological well-being. Participants were asked about the appropriateness and acceptability of the measures and procedures for recruitment and follow-up. In addition, the effects of the COVID-19 pandemic and lockdown measures on participants’ self-harm and general wellbeing were explored. All participating therapists were invited to be interviewed to explore their experiences of training, supervision and therapy delivery. These interviews were conducted by telephone and took place on three occasions: after initial therapy training, during therapy delivery, and after the final participant had completed therapy. Interviews were audio-recorded and transcribed.

Data from interviews with therapists was analysed thematically to identify positive components of therapist training and supervision, and perceived barriers to learning and therapy implementation. These findings were used to revise the training package for the subsequent RCT. A second analysis explored participants’ responses to understand the acceptability of the therapy. This explored how their experiences resonated with our initial theories as to how the intervention might work to enact change. Data collected from participant interviews were compared with data from the therapist that delivered their therapy in order to enhance our understanding of what worked for participants and therapists.

## Results

### Eligibility and recruitment

The study initially opened to recruitment in March 2020. However, recruitment was paused due to the COVID-19 pandemic. During this pause, one site dropped out due to staffing pressures from COVID-19 and a fourth site was recruited as a replacement. The study re-opened to recruitment in October 2020 and recruitment was completed in March 2021.

**Fig. 1:**
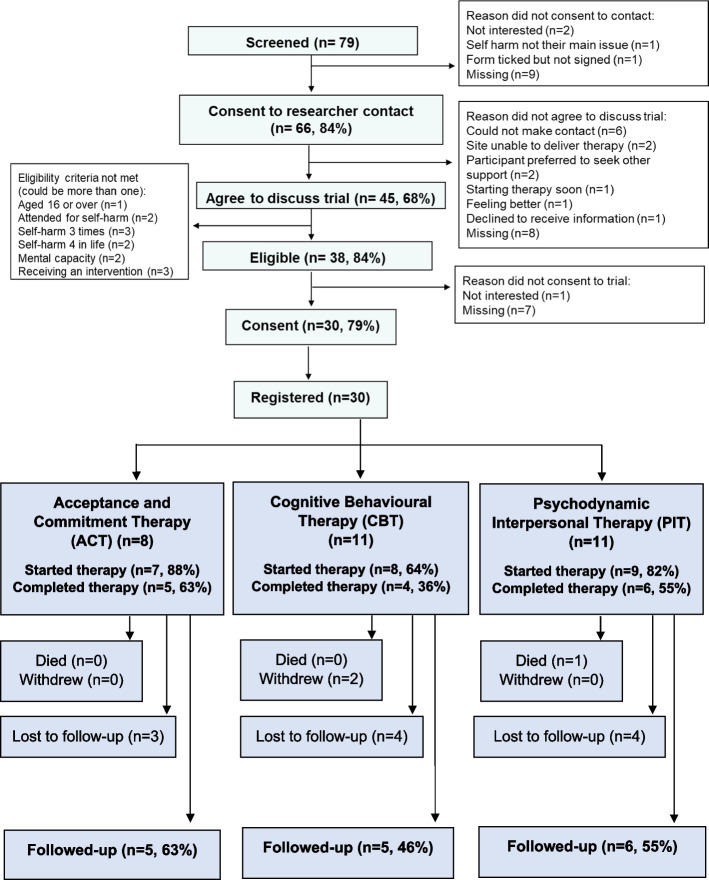
CONSORT diagram

Across three sites, 79 people were screened and of those, 30 (38%) participants were recruited (Fig. 1). Of those who consented to researcher contact, the main reason for not agreeing to the trial was that the researcher was unable to make contact. Of those who agreed to discuss the trial, the main reason for ineligibility was self-harm history (prior self-harm was not sufficiently high) or receiving a psychological therapy similar to the trial intervention.

Monthly recruitment primarily met the amber criteria. However, one site opened late and another site closed before the end of the recruitment period because they had reached their target.

Recruitment progression criteria: Average number of participants recruited per month.

**Fig. 2:**
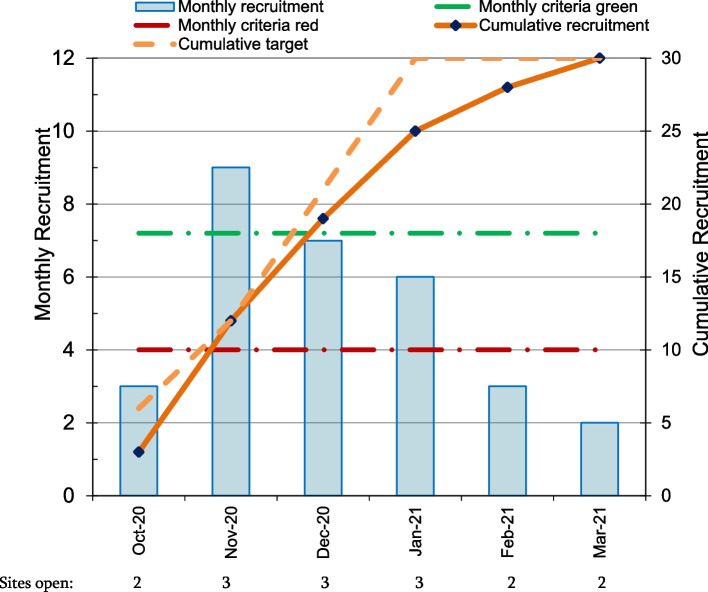
Recruitment graph

Recruitment progression criteria results: Mean monthly recruitment was 5 over the whole 6 months (amber). Monthly recruitment was ≥ 6 participants per month (7.3 participants per month on average, in the green range) when all 3 sites were open (Fig. 2).

### Baseline characteristics

The majority of participants were female, young adults, with a median age of 22 years. Of 30 participants, 27 (90%) were White British. Most participants (70%) had received therapy prior to being in the study. There was a wide range in the level of previous self-harm, with the number of times self-harmed in the previous 12 months ranging from 3 to 300 (Table 2).

**Table 2:** Baseline participant characteristics

	**Total (** ***N* = 30)**
**Site**	
Site 1	2
Site 2	15
Site 3	13
**Age (years)**	
Median (range)	22 (18, 58)
**Gender**	
Male	5 (16.7%)
Female	23 (76.7%)
Non-binary	1 (3.3%)
Prefer not to say	1 (3.3%)
**Ethnicity**	
White British	27 (90.0%)
Other	3 (10.0%)
**Sexual orientation**	
Straight/Heterosexual	16 (53.3%)
Other/Prefer not to say	14 (46.7%)
**Presenting self-harm method**	
Self-injury	12 (40.0%)
Self-poisoning	11 (36.7%)
Both	7 (23.3%)
**Number of self-harm episodes in the last 12 months**	
Median (range)	14 (3, 300)
**Times attended hospital as a result of self-harm in last 12 months**	
Median (range)	2 (1, 30)
**Currently on psychotropic medication/s**	26 (86.7%)
**Experience of abuse in their lifetime:**	
Physical abuse	12 (40.0%)
Sexual abuse	17 (56.7%)
Emotional abuse	18 (60.0%)
**In their lifetime:**	
Received talking therapy for mental health previously	21 (70.0%)
Received treatment for a mental health problem as an inpatient psychiatric unit and/or day patient psychiatric unit	8 (26.7%)

### Intervention acceptability

Of the 30 registered participants, 23 (77%) attended at least one therapy session of whom 15 (50% of all registered) completed therapy and attended all agreed sessions (Fig. 1).

Amongst the 8 who started but did not complete therapy, 1 participant stopped therapy based on the therapist’s decision since the participant was admitted to hospital as an inpatient, 1 participant died and the remaining 6 participants stopped therapy due to participant choice.

One participant died by suicide and therefore did not complete therapy. A full investigation by the research team and local NHS concluded that it was unrelated to the trial or the therapy; confirmed at the Coroner’s inquest.

Across all 30 registered participants, eight (27%) participants formally withdrew from therapy; four withdrew before starting therapy and four withdrew after attending between 2 and 5 sessions. Reasons for participants’ withdrawal from therapy were because they felt worse or not suited to therapy (*n* = 4); accepted another intervention (*n* = 1); did not feel well enough (*n* = 1); did not want contact with the local mental health team (*n* = 1) or due to an eligibility violation (*n* = 1).

**Fig. 3:**
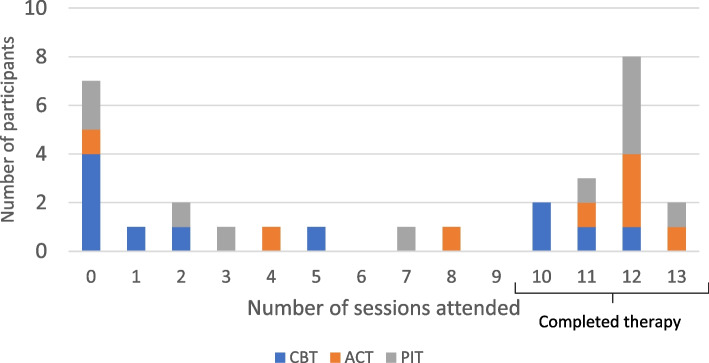
Number of sessions attended by therapy

Session attendance was similar across the three therapies (Fig. 3). A higher number of participants withdrew from CBT before beginning therapy (*n* = 3); however, this was not due to the specific therapy assigned in any of the 3 participants.

Intervention acceptability progression criteria: Proportion of registered participants attending their 1 st treatment session.

Intervention acceptability progression criteria results: 23 of 30 participants attended their first session. This is 77% of registered participants, which meets the green criteria.

### Intervention delivery

Fidelity to the common safety components was achieved for 78% of participants (*n* = 18/23), and FReSH START components were fully or partially addressed for 78% of participants (*n* = 18/23). Therapy-specific fidelity was variable: 40% for CBT, 50% for ACT and 100% for PIT. Therapists demonstrated warmth, rapport and empathy during the first session for the majority of participants (Table 3).

**Table 3:** Fidelity results

	**CBT**	**ACT**	**PIT**	**Overall**
Total	7	7	9	23
**Fidelity to safety components**	6 (85.7%)	7 (100.0%)	5 (55.6%)	18 (78.3%)
**FReSH START components fully addressed**	7 (100.0%)	4 (57.1%)	5 (55.6%)	16 (69.6%)
**FReSH START components partially addressed**	0 (0.0%)	2 (28.6%)	0 (0.0%)	2 (8.7%)
**Overall fidelity to safety and FReSH START components (4 out of 6 components)**	7 (100.0%)	6 (85.7%)	5 (55.6%)	18 (78.3%)
Number assessed for therapy–specific fidelity	5	6	6	
**Therapy-specific fidelity**	2 (40.0%)	3 (50.0%)	6 (100.0%)	
Number assessed for SPRS in first session*	7	7	9	23
Conveying warmth*				
Yes (SPRS ≥ 4)	5 (71.4%)	4 (57.1%)	7 (77.8%)	16 (69.6%)
No (SPRS < 4)	2 (28.6%)	3 (42.9%)	1 (11.1%)	6 (26.1%)
Missing	0 (0.0%)	0 (0.0%)	1 (11.1%)	1 (4.3%)
Rapport*				
Yes (SPRS ≥ 4)	6 (85.7%)	3 (42.9%)	7 (77.8%)	16 (69.6%)
No (SPRS < 4)	1 (14.3%)	4 (57.1%)	1 (11.1%)	6 (26.1%)
Missing	0 (0.0%)	0 (0.0%)	1 (11.1%)	1 (4.3%)
Empathy (SPRS ≥ 4)*				
Yes (SPRS ≥ 4)	6 (85.7%)	6 (85.7%)	8 (88.9%)	20 (87.0%)
No (SPRS < 4)	1 (14.3%)	1 (14.3%)	0 (0.0%)	2 (8.7%)
Missing	0 (0.0%)	0 (0.0%)	1 (11.1%)	1 (4.3%)

*Note: SPRS is Sheffield Psychotherapy Rating Scale. Warmth, rapport and empathy are rated on: 1—Not at all, 2, 3 – Some, 4, 5—Quite a lot, 6 and 7—Considerable amount. The initial session for 1 participant could not be rated due to issues with audio file formatting

Although fidelity to the self-harm components was mixed and most therapists introduced the concept that self-harm may have a function, this was often a limited discussion: in some cases, the therapist would only briefly introduce the concept with little in depth exploration of what this meant for the participant. Further, therapists generally did not address the possibility that self-harm may have more than one function for the participant. For some, the discussion of functions was limited by a focus on risk management instead. Interviews suggested that allowing participants to lead the focus on sessions, meant that some who were uncomfortable discussing self-harm were able to avoid in-depth discussion.

A total of 29 therapists were trained and 15 delivered therapy to trial participants. After the pause due to COVID-19, one site with 6 trained therapists could no longer participate due to reduced capacity. Of the remaining 23 therapists, 4 (17%) withdrew from the study before delivering therapy to a participant because the therapist moved post (*n* = 3) or the therapist was on long-term medical leave (*n* = 1). No therapists withdrew after delivering therapy to a participant.

Intervention delivery progression criteria: Proportion of participants where the therapist achieved intervention fidelity in terms of safety and FReSH START components.

Intervention delivery progression criteria results: Intervention fidelity (FReSH START and safety components) was achieved for 18 of 23 participants who started therapy. This is 78% of those who started therapy, reaching the amber criteria.

### Follow-up and outcome measurement

The 6-month follow-up questionnaires were completed for 16 (53%) participants. Reasons for non-completion were due to: loss to follow-up in 11 (37%) participants, withdrawal in two (7%) participants and one (3%) participant died during the study.

Baseline characteristics were similar between participants who were followed up and those who were not (Additional File 3). Follow-up rates varied across the three sites (0% (0/2) in site 1, 47% (7/15) in site 2 and 69% (9/13) in site 3). Participants who completed follow-up attended more sessions on average than those who did not; however, five of the 15 participants who completed therapy did not return the 6-month questionnaire.

Of the 16 participants who completed the follow-up questionnaire, only one (6%) person reported needing help with the questionnaire, which they received from their partner/spouse. Items on the participant-reported outcome measures were well completed, with a maximum of only one missing item for the Beck Hopelessness Scale, and no missing items on the CORE-OM, PHQ-9 or SCS-R.

Responses to text messages were high in the first month (≥ 80%). However, this reduced to 52% by 6 months (Table 4). Participants who responded to the monthly text messages were more likely to return the 6-month questionnaire (Additional File 4).

**Table 4:** Response rates to monthly text messages

	**N sent**	**Responded to general health question**	**Respond to self-harm question**
Month 1	27	23 (85.2%)	22 (81.5%)
Month 2	25	17 (68.0%)	19 (76.0%)
Month 3	25	18 (72.0%)	17 (68.0%)
Month 4	25	14 (56.0%)	17 (68.0%)
Month 5	25	15 (60.0%)	16 (64.0%)
Month 6	25	13 (52.0%)	13 (52.0%)

Participants were contacted by text and phone call or by email depending on their stated preference. In almost all cases, participants were more responsive when they could see the number calling, and after receiving a message (answerphone or text) stating the purpose of the contact. Multiple attempts to make contact were required before a response was received, regardless of whether the participant eventually completed the follow-up questionnaires, and once contact was made, participants were usually open to completing the questionnaires.

Follow-up progression criteria: Proportion of registered participants completing the CORE-OM at 6 months.

Follow-up progression criteria results: 16 (53%) participants completed the CORE-OM at 6 months, which was in the red criteria.

### Statistical outcomes

Summary scores of participant-reported outcome measures (Table 5) were similar at baseline between those followed-up at 6 months and those who were not.

**Table 5:** Summaries of participant-reported outcomes

	Baseline (all participants, *n* = 30)	Baseline (of participants followed-up, *n* = 16)	6 months (*n* = 16)
**CORE-OM Total score**			
Mean (SD)	21.0 (5.73)	20.2 (5.92)	16.9 (8.27)
95% CI	(18.83 to 23.10)		(12.54 to 21.36)
Range	12.4 to 33.5	12 to 32	5.9 to 29.7
**CORE-OM Categories**			
Clinical (10 to 40)	30 (100.0%)	16 (100.0%)	12 (75.0%)
Non-clinical (Less than 10)	0 (0.0%)	0 (0.0%)	4 (25.0%)
**Reliable and clinically significant improvement (RCSI) on CORE-OM**			0 (0.0%)
**CORE-OM total change from baseline**			
Mean (SD)			− 3.2 (5.44)
95% CI			(− 6.13 to − 0.34)
Range			− 11.5 to 7.4
**Beck Hopelessness Scale total score**			
Mean (SD)	14.4 (4.80)	13.3 (4.70)	10.6 (6.41)
95% CI	(12.60 to 16.18)		(7.16 to 14.00)
Range	3.0 to 20.0	4.0 to 20.0	1.0 to 20.0
**PHQ-9**			
Mean (SD)	18.7 (5.23)	18.6 (5.49)	14.4 (7.90)
95% CI	(16.76 to 20.67)		(9.80 to 18.92)
Range	9.0 to 26.0	9.0 to 26.0	2.0 to 27.0
**SCS-R**			
Mean (SD)	59.2 (17.68)	59.0 (15.45)	66.3 (23.74)
95% CI	(52.56 to 65.77)		(53.66 to 78.96)
Range	22.0 to 101.0	35.0 to 90.0	28.0 to 99.0

Clinical Outcomes in Routine Evaluation–Outcome Measure (CORE-OM): Range from 0 to 40 with a higher score indicating higher levels of distress. Reliable and clinically significant improvement (RCSI) on the CORE-OM was defined as: change in CORE-OM of 5 or more points (reliable) and movement from the clinical range (≥ 10/40) to the non-clinical range (< 10/40) (clinically significant) [34–36]

Beck Hopelessness Scale (BHS): Range from 0 to 20 with a higher score indicating higher levels of hopelessness. Patient Health Questionnaire-9 (PHQ-9): Range from 0 to 27, with a higher score indicating higher levels of depression. Social Connectedness Scale—Revised (SCS-R): range from 20 to 120, with a higher score indicating more connectedness to others

*SD* Standard deviation, *CI* Confidence interval

Investigation into the ICC of the CORE-OM by therapist was not meaningful since there were only 1–2 participants per therapist in the analysis. The ICC by therapy was very small (< 0.01). The standard deviation in the CORE-OM at 6 months was 8.27, which was very similar to the value of 8 assumed in the sample size calculation for the definitive trial.

### Health economics outcomes

The health economics outcomes are summarised in Table 6 and reported in full in Additional File 5. In addition to the 14 participants who did not provide any follow up data, there was one missing entry for costs and HRQoL at baseline, and two missing entries for work absenteeism at follow up. Participants who were followed-up at 6 months had higher mean baseline HRQoL and higher mean costs compared to those who were not.

**Table 6:** Health economics outcomes summary

	Baseline (all participants, *n* = 30)	Baseline (of participants followed-up, *n* = 16)	6 months (*n* = 16)
**Health-related quality of life (HRQoL)**			
Mean (SD)	0.69 (0.21)	0.75 (0.20)	0.75 (0.19)
95% CI	0.61, 0.77	0.65, 0.86	0.65, 0.85
Range	0.10, 0.94	0.16, 0.94	0.32, 0.95
* N*	29	16	16
* N* missing	1		
**Quality adjusted life years (QALYs)** ^**1**^			
Mean (SD)	NA	NA	0.38 (0.09)
95% CI	NA	NA	0.33, 0.42
Range	NA	NA	0.12, 0.45
* N*	NA	NA	16
* N* missing	NA	NA	
**Healthcare Costs** ^**2**^			
Mean (SD)	£278.40 (£235.20)	£294.65 (£269.94)	£232.95 (£198.63)
95% CI	£188.94, 367.87	£150.82, £438.49	£127.11, £338.80
Range	£22.23, £924.08	£33.15, £924.08	£0.00, £655.10
N	29	16	16
N Missing	1		
**Absenteeism**			
Mean (SD)	£77.79 (£180.29)	£101.07 (£229.61)	£23.33 (£55.89)
95% CI	£10.46, £145.11	£0.00, £223.42	£0.00, £55.59
Range	£0.00, £868.51	£0.00, £868.51	£0.00, £180.95
* N*	30	16	14
* N* Missing	0		
**Out of pocket expenses**			
Mean (SD)	£34.38 (£60.36)	£48.56 (£73.67)	£35.11 (£78.44)
95% CI	£11.84, £56.92	£9.30, £87.82	£0.00, £76.90
Range	£0.00, £200.00	£0.00, £200.00	£0.00, £295.00
* N*	30	16	16
* N* Missing	0		

*SD* standard deviation,

*CI* confidence interval

^1^QALYs calculated across previous 6 months

^2^Healthcare costs included community care and outpatient appointments. Excludes medication costs which were not collected in the baseline questionnaire

Intervention costs (per participant) for ACT, CBT and PIT were £986, £929 and £957 respectively, and were highly dependent on the method to incorporate training and supervision costs (Additional File 5).

Medication costs (baseline) and participant travel costs (baseline and follow up) were not collected during the feasibility study, amendments have been made to questionnaire items to ensure they are collected in the definitive trial.

### Qualitative interviews

Participants found the therapy as a whole acceptable and perceived benefits from it even if individual therapeutic components were not quite right for them. Benefits included therapy as a means of preparing them for future interventions, improved relationships with NHS staff and as an opportunity to open up to a trusted person who was willing to listen. These positive experiences were often driven more by a trusting and warm relationship with their therapist than by therapy-specific components. Some participants credited this relationship for remaining in therapy for 12 weeks. Therapists also felt positively about the therapy and the opportunity to offer a longer term relationship with participants. Although most felt prepared to commence therapy, training was regarded as an initial component, with supervision essential to support their confidence.

Patients’ past experience of therapy, their perceptions of the therapist, as well as wider life factors, interacted with therapist behaviours to influence their engagement with the therapy. However, context was also key: support from clinical teams could be mitigated by service pressures which made it difficult to protect time for regular session delivery. Some therapists found that this lack of continuity could reduce participants’ engagement.

The therapeutic component that addresses self-harm as having a function was not always discussed during initial sessions. In some cases, there was a strong focus on safety planning. Although mental health liaison nurses were recruited for their ability to manage risk, we found that some therapists had difficulty shifting the session focus away from a ‘service provision’ mode and towards more in-depth discussion of feelings.

These findings indicated a need for emphasis on supervision and the introduction of a therapist readiness check to increase their confidence; working with study site teams to emphasise the need to protect therapist time to deliver sessions.

### Overall progression criteria

The results of the progression criteria are shown in Table 7. Participant recruitment was within the amber range; however, average monthly recruitment met the green target during the period when all three sites were open. Intervention acceptability was high. Intervention delivery was acceptable but requires modifications for the definitive trial. Follow-up rates were poor overall but highly variable across sites and require major changes to proceed to the definitive trial. Loss to follow-up could not be explained by baseline characteristics but was associated with low therapy attendance and SMS response rates.

**Table 7:** Results of progression criteria

	Red	Amber	Green (95% CI)	**Outcome**	**Red/Amber/Green result**
Recruitment—Progression criteria 1: Average number of participants recruited per month	< 4	4–7	> 7	Monthly recruitment was ≥ 7 when all 3 sites open Mean monthly recruitment was 5 over the whole 6 months	Amber: 5 participants per month (Amber to green during period when all 3 sites open)
Follow-up—Progression criteria 2: Proportion of registered participants completing the CORE-OM at 6 months	< 60%	60–75%	> 75% (95% CI: 59.5%, 90.5%)	16 of 30 participants completed the CORE-OM at 6 months	Red: 53%
Intervention delivery—Progression criteria 3: Proportion of participants where the therapist achieved intervention fidelity in terms of safety and FReSH START components	< 60%	60–80%	> 80% (95% CI: 65.7%, 94.3%)	Overall: 18 of 23 who started therapy Overall: 18 of 30 registered Acceptance and Commitment Therapy (ACT): 6 of 7 who started therapy Cognitive Behavioural Therapy (CBT): 7 of 7 who started therapy Psychodynamic Interpersonal Therapy (PIT): 5 of 9 who started therapy (1 missing)	Amber: Overall 78% (of those who started therapy)
Intervention acceptability—Progression criteria 4: Proportion of registered participants attending their 1st treatment session	< 50%	50–70%	> 70% (95% CI: 53.6%, 86.4%)	23 of 30 participants attended their first session	Green: 77%

## Discussion

### Summary of changes for the definitive trial

In this feasibility study, we were able to successfully deliver training to mental health professionals, recruit participants and deliver the intervention in a way that is acceptable to participants, despite disruption due to the pandemic. On the basis of the results of this feasibility study, we plan to proceed with the definitive RCT. There will be no fundamental changes to the planned study design for the definitive trial and changes to the study processes will focus on improving follow-up, incorporating routinely collected data, and improving intervention fidelity. Training has also been modified so that it will be delivered fully online instead of face-to-face.

In the definitive trial, we plan to offer online questionnaire completion via REDCap, as well as optional postal or researcher-supported telephone completion, to improve completion rates. We will collect additional contact details for a friend or relative of the participant, where the participant consents to this. Online questionnaires and the additional contact should help to make it easier to contact participants who may change address frequently.

We will provide £20 vouchers to participants for completion of 6- and 12-month questionnaires, which is an evidence-based strategy to improve retention [37]. To further improve retention, we will include a letter from the Lived experience group with the follow-up questionnaire to emphasise the importance of continued participation in the trial, regardless of the participant’s treatment allocation or outcomes; the letter content will be informed by behaviour change strategies [38]. The definitive trial will use routinely collected data including Hospital Episode Statistics via NHS digital data to obtain outcomes on hospital attendance due to self-harm (and other reasons), allowing data collection for participants lost to follow-up who do not return participant-reported questionnaires [39]. We will also incorporate the complete routinely collected data as a set of conditioning variables in the multiple imputation procedures adopted to impute missing data for the primary endpoint and other outcome variables in the main trial. This makes the necessary assumption of data Missing At Random in multiple imputation more tenable as we will be able to condition on hospitalisations. The training for all three therapies has been amended based on feedback during the feasibility study. Fidelity to FReSH START components was good in the CBT and ACT therapies but required improvement in the PIT therapy. This is likely to be due to the FReSH START components involve a functional analysis of self-harm behaviour which is not usually a part of the PIT approach. The PIT therapist training has subsequently been adapted to focus more on the FReSH START components.

Therapy-specific fidelity was excellent in PIT but required improvement in the ACT and CBT therapies. Nearly all the therapists in the study were generally new to delivering any psychological therapy. They may have found delivery of ACT particularly challenging because it includes counter-intuitive ideas and methods. For example, contrasting with usual practice in mental health services where the treatment focus is on pragmatic ways for reducing distress, ACT primarily encourages engagement in activity that the participant sees as life enhancing. This goal sometimes requires therapists to suggest behaviours like increased willingness to have challenging thoughts and emotions etc. Consequently, ACT and CBT therapist training has been extended for the main trial, to include additional time to focus on key aspects of the model and intervention methods where fidelity was poor.

To assess the success of these changes in the definitive RCT, we plan to conduct extra fidelity checks when each therapist treats their first participant. We will incorporate a therapist readiness check at the end of therapist training to ensure therapists are sufficiently knowledgeable prior to treating their first participant and monitor attendance for each therapy to check for high rates of withdrawal across any of the three therapies.

As the main trial will incorporate the usual care comparator arm, the primary analysis will compare the FReSH START arm (all 3 modalities combined) with the usual care arm. Further details are provided in the published protocol for the definitive trial [40].

### Impact of COVID-19

Study site discussions and interviews with therapists indicated multiple direct impacts of Covid-19 on delivery of the intervention and research components, including:

- Delays due to pause on recruitment
- General strain on NHS departments, staff shortages, therapist capacity, limited availability of rooms to deliver therapy, competing priorities, and general staff morale. This affected the numbers of potential participants being screened by liaison staff and also impacted on capacity to release therapists to deliver therapy. Interviews indicated that liaison teams were supportive of the study but sometimes found it difficult to translate this into practical support due to limited resources.
- Changes to the nature and number of presentations for self-harm during the pandemic: attendances fluctuated with a reduction in all self-harm attendances at times, and a greater number of more severely unwell patients at other times, leading to fewer eligible patients.
- Changes to physical location of liaison teams and modes of interaction with patients: in some sites, liaison teams were relocated to different buildings requiring telephone assessments rather than face to face, or patients needing to leave ED for an assessment
- Adapting training to online delivery format, general disruption to training
- Adapting therapy to be delivered in an online format if needed/desired: this made therapy delivery more flexible but also presented technical challenges for therapists
- Participants (e.g. students studying remotely at times) leaving their Trust catchment area and impacting on risk management

### Limitations

The limitations of this feasibility study are that it was conducted on a relatively small sample of 30 participants across 4 sites. This was a single-arm study where all participants received therapy. Therefore, we did not test the process of randomising participants to receive therapy or usual care. The use of a usual care arm in the definitive trial could impact on recruitment and retention rates.

Recruitment began in October 2020 following a pause due to the COVID-19 pandemic when health services remained under high pressure, and this may have been a turbulent time for patients. This may not be reflective of the situation before the COVID-19 pandemic when the study was planned, however it should be similar to the setting in future when the follow-on definitive trial will be conducted.

### Implications for future research

This study has shown that participants who repeatedly self-harm can be recruited into a feasibility trial of psychological therapies. Therapy, therapist training and supervision can all be delivered both face-to-face and remotely.

The results demonstrate the value of conducting a feasibility study to enhance trial processes. This allowed the study team to make changes to the therapist training aimed at improving therapist fidelity to the intervention. Follow-up processes will also be enhanced in the definitive trial with the aim of improving retention. This knowledge from the feasibility study should allow the full-scale definitive trial to evaluate an intervention that matches more closely what was originally planned and to assess outcomes in a larger and more generalisable sample of participants.

## Conclusions

During times of difficulty due to pressure from COVID-19, we were able to train mental health professionals and recruit participants into a trial of modified psychological therapies for people who repeatedly self-harm. We successfully delivered the intervention with high levels of attendance at therapy sessions. However, participant follow-up was challenging and intervention fidelity requires improvement. This study demonstrates the feasibility of such a trial with modified study processes to improve participant retention and intervention fidelity.

## Supplementary Information

Additional file 1. Details of three adapted approaches and TIDieR tables

Additional file 2. Details of item-level imputation of patient-reported outcome measures

Additional file 3. Baseline characteristics comparing those who were followed-up or not

Additional file 4. Text message response rate by whether returned questionnaire or not

Additional file 5. Health Economics Costs Analysis

## Data Availability

Data and materials are available upon reasonable request. Requests for data and materials should be initially made to the Chief Investigator, Elspeth Guthrie (E.A.Guthrie@leeds.ac.uk).

## Abbreviations

ACT: Acceptance and commitment therapy
ACT-FM: ACT Fidelity Measure
BHS: Beck Hopelessness Scale
CBT: Cognitive behavioural therapy
CI: Confidence interval
CORE-OM: Clinical Outcomes in Routine Evaluation—Outcome Measure
CTS-R: Cognitive Therapy Scale (Revised)
ED: Emergency Departments
FReSH START: Function REplacement in repeated Self-Harm: Standardising Therapeutic Assessment and the Related Therapy
ICC: Intra-cluster correlation coefficient
ITT: Intention-to-treat
PHQ-9: Patient Health Questionnaire-9
PIT: Psychodynamic interpersonal therapy
RCSI: Reliable and clinically significant improvement
SCS-R: Social Connectedness Scale – Revised
SD: Standard deviation
SPRS: Sheffield Psychotherapy Rating Scale
UC: Usual care
