# Supplementary material for: Results of a feasibility study of the FReSH START intervention to improve quality of life and other outcomes in people who repeatedly self-harm (Function REplacement in repeated Self-Harm: Standardising Therapeutic Assessment and the Related Therapy)
Source: Pilot Feasibility Stud. 2025 May 15;11:67. doi: 10.1186/s40814-025-01644-2 (PMC12080260; doi:10.1186/s40814-025-01644-2)
Supplement: Supplementary file 1 — Additional file 1. Details of three adapted approaches and TIDieR tables [file 40814_2025_1644_MOESM1_ESM.pdf]

Additional file 1: Details of three adapted approaches and TIDieR tables

Additional file 1.1 Details of the three adapted approaches

The specific details of the three adapted approaches are described below:

Cognitive Behaviour Therapy (CBT)

CBT draws on cognitive and behavioural approaches to understanding emotional distress. Cognitive approaches see the way we think about events as influencing our emotional reaction to them. Fundamental beliefs (a schema) once activated give rise to negative thoughts, which maintain emotional difficulties through a series of feedback loops, including behaviours. Behavioural approaches involve trying to understand the pattern of relationships between behaviours and emotional responses in terms of the function of the behaviour and then seeking to introduce new patterns of behaviour. The two theoretical approaches inform CBT treatment techniques, though different types of CBT may place more or less emphasis on one theoretical approach.

Acceptance and Commitment Therapy (ACT)

ACT is a newer form of cognitive behaviour therapy that aims to engender a quality called 'psychological flexibility', which can be defined as: "…the capacity to persist or to change behaviour in a way that includes conscious and open contact with thoughts and feelings (openness), appreciates what the situation affords (awareness), and serves one's goals and values (engagement)." McCracken & Morley, P225

Consequently, in ACT, within an equal, kind and empowering therapeutic relationship, a clinician will offer a range of therapy methods and techniques to enhance psychological flexibility. Depending on the person, this might include mindfulness practice, values-elicitation, self-compassion and/or perspective-taking exercises etc. For example, where indicated, a clinician might help a person connect with their own over-arching goals and values by asking questions about what or whom is important in their life, then helping them to consider ways to match their behaviours with their values.

Psychodynamic Interpersonal Therapy (PIT):

This is a psychodynamic form of therapy which aims to manage feelings in the context of interpersonal relationships. It focuses upon interpersonal problems or ways of relating which may underpin symptomatic or problem scenarios. There is a strong focus on developing a strong therapeutic alliance from which interpersonal problems can be identified and solved. The different components of the model are as follows: 1) focus on feelings 2) encourage the client to stay with feelings 3) explore what associated thoughts, images, memories come to mind 4) explore links or patterns in interpersonal relating that are problematic 5) acknowledge these problematic patterns 6) test out new ways of behaving both in the session with the client and in personal relationships outside. A goodbye letter is given to the client at the end of the therapy to summarise the work.

Additional file 1.2 TIDieR tables for the 3 adapted approaches

TIDieR Template for Intervention Description and Replication

Psychodynamic Interpersonal Therapy – Self-Harm (PIT-SH)

e	Item	
		Other † (details)
	**BRIEF NAME**	
**1.**	Provide the name or a phrase that describes the intervention.	Psychodynamic Interpersonal Therapy-Self-harm (PIT-SH)
	**WHY**	
**2.**	Describe any rationale, theory, or goal of the elements essential to the intervention.	Psychodynamic Interpersonal Therapy (also called the Conversational Model of Therapy) was developed in the 1970s and draws on both psychodynamic and interpersonal principles, using jargon free language.<br><br>It focuses upon interpersonal problems or ways of relating which may underpin symptomatic or problem scenarios. There is a strong focus on developing a strong therapeutic alliance from which interpersonal problems can be identified and solved. The different components of the model are as follows: 1) focus on feelings 2) encourage the client to stay with feelings 3) explore what associated thoughts, images, memories come to mind 4) explore links or patterns in interpersonal relating that are problematic 5) acknowledge these problematic patterns 6) test out new ways of behaving both in the session with the client and in personal relationships

		outside. The therapist uses specific actions which facilitate the expression of feelings or the deepening of the therapeutic alliance. These include: making statements rather than asking questions; using a gentle negotiating style; using conversational approach; picking up cues; staying with feelings in the ‘here and now’ and exploring the links of those feelings to relationships past and present.<br><br>PIT can be used in a brief or long term format and has been evaluated in a variety of different formats, ranging from 3 session (2 plus one) , four session, 8 sessions, 12 sessions and 12-24 months twice weekly. PIT can be delivered by a range of professionals including psychiatrists, clinical psychologists, social workers, GP counsellors, liaison nurses.<br><br>**Application of psychodynamic-interpersonal therapy to managing repeated self-harm**<br><br>PIT has been evaluated for the treatment of self-harm in a randomized controlled trial of 119 patients who were randomized to 4 sessions of PIT delivered by liaison nurse plus usual care versus treatment as usual alone (Guthrie et al, 2001). There was a reduction in reported self-harm in the six months post-treatment in the patients who received PIT in comparison with the usual treatment group. Approximately 50% of the patients who participated in the trial had a prior history of self-harm.<br><br>Following this study, an NHS treatment service, called the SAFE-team was established to deliver PIT to patients who had self –harmed (both repeaters and first episode patients). The service was based in Manchester and ran for over 15 years.

		SAFE-PIT is the modified version of PIT that was employed by the service and is specifically tailored for people who SH. There is a focus on the most recent self-harm episode and an attempt to get the client to re-experience his/her feelings at the time of the self-harm behavior, to stay with those feelings and see what thoughts, images emerge. A goodbye letter is given to the client at the end of the therapy to summarise the work and re-enforce positive change.
	**WHAT**	
**3.**	Materials: Describe any physical or informational materials used in the intervention, including those provided to participants or used in intervention delivery or in training of intervention providers. Provide information on where the materials can be accessed (e.g. online appendix, URL).	Materials: 1) Generic FReSH START Manual which includes information about self-harm, results of the FReSH START research in the first part of the programme including findings from a systematic review of what service users find helps them to stop self-harming and a Q study to understand the underlying functions of self-harm. The most common functions of self-harm described by services users are described in the manual to help guide the therapist explore client function in the initial sessions. The manual also provides guidance on risk assessment and safety planning, and the structure of therapy. Includes an appendix comprising standardised documents for risk assessment safety planning, and assessing the functions of self-harm.<br><br>2) PIT-specific FReSH START manual which describes the principles of PIT and the rationale for its application to helping people who self-harm; explains and exemplifies basic PIT therapeutic skills (active listening, picking up cues, making statements). Describes and exemplifies basic PIT therapy techniques (staying with feelings in the here and now, using metaphor to deepen understanding, developing forms of feeling; includes example of good bye letters, and PIT-fidelity.<br><br>3) PIT-specific fidelity assessment criteria rating the degree to which therapists adhered to and applied the PIT principles to their sessions will be used to assess ‘trial readiness’. These criteria are rated on a 1-7 scale and focused around the previously mentioned PIT therapeutic skills and techniques.

		<u>Patient/Participant facing materials</u><br><br>Participants will receive a detailed personal goodbye letter which describes their experience of therapy, postivie changes that have been made and positive ways to manage distressing feelings in the future.<br><br><u>Clinician training resources</u><br><br>Training resources include: 1) the aforementioned clinician manual; 2) a PowerPoint slide deck for the two-day training session; 3) Website hosting videos of role plays and manual.
**4.**	Procedures: Describe each of the procedures, activities, and/or processes used in the intervention, including any enabling or support activities.	There are 12 sessions (with two possible booster sessions) in FReSH START. These are divided up into four stages:<br><br>1) <u>Sessions 1-3: Assessment</u><br>• Examine the function of self-harm according to the FReSH STARTassessment.<br>• Conduct a risk assessment.<br>• Discuss and develop a safety plan if it is relevant and the participant does not have one and would like to have one.<br>• Focus on engagement of the client and awareness of any signs or cues that signal ambivalence or a likelihood of dropping out.<br>• Address any ambivalence early on.

		• Focus on most recent SH episode. Feelings and any warded off feelings and thoughts. Link to relationships or patterns of behaviour.<br>• Emphasis on getting to know the person as opposed to lots of facts about them. Identify their values and what matters to them as a person.<br>• Emphasis upon developing a relationship with the client and generating a feeling of being understood by the client.<br>• Identify areas the client would like to change. Awareness of barriers to change any common therapeutic problem scenarios.<br><br>Session 4-9<br><br>• Further deeper exploration of the problem area and links to interpersonal relationships<br>• Current social support network, family relationships and past important relationships.<br>• Exploration of patterns in relationships<br>• Focus upon change behaviours in relationships. Both in the session with the therapist and outside.<br>• Managing feelings in the here and now.<br><br>Session 10-12<br><br>• Development of goodbye letter.<br>• Discuss in supervision whether booster sessions required<br>• Focus on ending so sessions can be brought to an end in a constructive and positive way for the client acknowledging difficulties or any distress that results from the ending.<br>• Address any problems or difficulty with endings.

		• Focus upon positive change but also any future problems which may arise for the client in the immediate aftermath of therapy.<br>• Rehearse how these can be managed positively.
	**WHO PROVIDED**	
**5.**	For each category of intervention provider (e.g. psychologist, nursing assistant), describe their expertise, background and any specific training given.	The intervention will be delivered by liaison mental health professionals working with self-harm, most commonly nurses. Other mental health staff who work in acute mental health services may also be considered. Potential therapists will have prior expertise in the risk assessment and management of service users who present with self-harm. Potential therapists will be embedded within acute mental health services with access to a Consultant Psychiatrist or nominated deputy to help manage/discuss risk issues. There is no requirement for previous training in psychological therapy, but staff who have a prior psychological therapy training can be included. Potential therapists must have pre-existing good interpersonal skills. Potential therapists will receive online training in the FReSH START assessment and PIT for self-harm. This will consist of 6-8 on line sessions of theory, didactic teaching, small group work and role plays. All potential therapists will undergo a trial readiness assessment at the end of training to assess their basic therapeutic skills. All therapists will receive ongoing regular supervision (at least one session of supervision every two weeks) from an experienced PIT therapist whilst they are delivering treatment.
	**HOW**	
**6.**	Describe the modes of delivery (e.g. face-to-face or by some other mechanism, such as internet or telephone) of the intervention	The intervention is one to one and can be provided face to face or via video link or telephone.<br><br>If the intervention is delivered face to face it will be conducted with appropriate social distancing and appropriate degrees of personal protective equipment.<br><br>All sessions will be audio-recorded and encrypted using a suitable portable device.

	and whether it was provided individually or in a group.	
	**WHERE**	
**7.**	Describe the type(s) of location(s) where the intervention occurred, including any necessary infrastructure or relevant features.	Mental Health Trust clinical offices or Acute Hospital Trust offices or remote delivery.
	**WHEN and HOW MUCH**	
**8.**	Describe the number of times the intervention was delivered and over what period of time including the number of sessions, their schedule, and their duration, intensity or dose.	There will be 12 sessions of weekly therapy, 50 minutes- 1 hour duration. Completion of therapy is defined as either 12 sessions or a mutually agreed ending.<br>2 additional booster sessions are allowed at the discretion of the therapist and their supervisor.<br>These additional sessions will be delivered by telephone.<br>All sessions including booster sessions must be completed within six months of randomisation.
	**TAILORING**	

**9.**	If the intervention was planned to be personalised, titrated or adapted, then describe what, why, when, and how.	The intervention has stages and therapists must adhere to a PIT approach and use PIT skills accordingly. However, the content of sessions is tailored to each participant and the emotional difficulties or feelings they bring to the session.<br><br>Further, a dominating life problem (e.g. loss of home, end of a relationship, issue with the authorities) may mean that the focus of therapy is most appropriately focused on managing this issue. Here, a therapist will focus simply on helping the participant to be aware of their thoughts and feelings in order to make effective choices (i.e. do things that maintain well-being and continue to enrich) in the presence of a significant stressor. In such an instance a therapist may decide to simply use the basic (PIT) therapy skills as the participant reviews their week, discusses the ongoing stressor and plans for the week ahead.
	**MODIFICATIONS**	
**10.**[‡]	If the intervention was modified during the course of the study, describe the changes (what, why, when, and how).	During the feasibility study, the mode of therapy delivery was widened so the therapy could be delivered face to face (with appropriate social distancing), or by video conferencing or by telephone.
	**HOW WELL**	
**11.**	Planned: If intervention adherence or fidelity was	For all participants who start therapy, the initial 1-3 sessions will be rated for fidelity to safety and FReSH START components.

	assessed, describe how and by whom, and if any strategies were used to maintain or improve fidelity, describe them.	One additional session of each participants subsequent sessions will be rated for fidelity to therapy specific components. The session reviewed will be randomly selected from the available sessions for each participant.<br><br>Startup, M., Shapiro, D.A. Therapist treatment fidelity in prescriptive vs. exploratory psychotherapy. Br J Clin Psychol 1993; 32 (4) :443-56.
**12.‡**	Actual: If intervention adherence or fidelity was assessed, describe the extent to which the intervention was delivered as planned.	The therapy-specific components for PIT were delivered as planned in all reviewed sessions. However, the FReSH START components and safety components were often not fully addressed. The therapist training will be adapted in the definitive trial to focus on the FReSH START components.

TIDieR Template for Intervention Description and Replication

Acceptance and Commitment Therapy (ACT) in the context of Self-Harm

**Item number**	**Item**	Other † (details)
	**BRIEF NAME**	

**1.**	Provide the name or a phrase that describes the intervention.	A brief Acceptance and Commitment Therapy (ACT) approach to enhancing life-enriching activity in the context of self-harm designed to be delivered by new psychological therapists
	**WHY**	
**2.**	Describe any rationale, theory, or goal of the elements essential to the intervention.	ACT is an acceptance-based behaviour therapy that has been developed over the past 20 years. Unlike traditional CBT, ACT does not explicitly aim to reduce distress or to change negative thoughts. Instead, ACT aims to enable effective action in the presence of competing sources of psychological influence (this might include emotions and thoughts, sensations, habits). The focus of ACT is thus on enhancing psychological flexibility, which consists of three components:"…the capacity to persist or to change behaviour in a way that 1) includes conscious and open contact with thoughts and feelings (openness), 2) appreciates what the situation affords (awareness), and 3) serves one's goals and values (engagement)" (15), p. 225.).<br><br>ACT can be delivered in a range of formats. In routine NHS clinical practice, ACT for individuals tends to comprise six to twelve hour-long sessions with a suitably qualified clinician, usually a clinical psychologist, or a CBT therapist.<br><br>ACT includes a range of methods for engendering psychological flexibility. The therapist will explore the participant's 'values': freely chosen qualities of ongoing action that serve as intrinsically reinforcing means to co-ordinate future behaviour. (Examples might be performing as a nurturing parent, or creativity in work.) Participants are then encouraged consciously to initiate actions that support these values: e.g. spending time with children, disclosing emotions and opinions, etc.. To assist this committed action, the therapist facilitates development of the openness and awareness aspects of psychological flexibility. This entails teaching skills

		derived from mindfulness practice, or defusion (learning to notice the actions of the mind as separate from the self), perspective-taking and self-compassion exercises.<br><br>**Application of acceptance and commitment therapy to managing repeated self-harm**<br><br>Unlike treatments used most regularly in the context of self-harm (CBT, DBT etc.), the focus of ACT is not on learning to control painful emotion or even necessarily to stop self-harm as the primary goal. It instead teaches participants to take a broader perspective on their behaviours (including self-harm); to notice whether their choices and actions are taking them closer to, or further away from, their values in the longer-term. A range of newer methods for approaching emotions and thoughts that interfere with meaningful activity – including acceptance of emotions and self-compassionate perspectives – are then suggested to participants, and they may experiment with these between sessions.<br><br>Nonetheless, ACT may be an apt strategy to intervene where positive, and not just negative, reinforcement is maintaining self-harm. For example, mastery functions of self-harm may include positive reinforcement via consequential pleasurable thoughts (success, in control), emotions (pleasure, vitality, confidence, relaxation etc.) or responses from others/systems. To intervene, in ACT participants explore their values with the clinician, to get a sense of what is personally meaningful to them (e.g. being present to their children, supporting others, etc.). From here, ways to notice self-harm behaviours from a broader perspective and to see their effectiveness – i.e. whether interfering with, or helping them to make progress on, their values – is encouraged. The influence of competing sources of psychological influence (here reinforcing positive feelings, thoughts, behaviours) can also be reduced in several ways: The therapist will help the participant learn to notice, distance from, and open-up to,

		positive thoughts and feelings that come with self-harm. For example, it could be that learning to distance from thoughts regarding mastery, as opposed to becoming entangled in them, helps to reduce the influence of these phenomena over meaningful activity; or that exploring ways to open up to, or to notice, the pull of pleasurable feelings helps to reduce their influence.<br><br>In addition, we think that ACT is a pragmatic choice of treatment because it is already widely-used within NHS services. It is probably the most practiced psychological therapy in NHS chronic pain services, and commonly used in NHS clinical health psychology and adult mental health services. There are various schemes (e.g. pathfinder scheme in Sheffield) where ACT is being taught to IAPT clinicians.
	**WHAT**	
**3.**	Materials: Describe any physical or informational materials used in the intervention, including those provided to participants or used in intervention delivery or in training of intervention providers. Provide information on where the materials can be accessed (e.g. online appendix, URL).	<u>Clinician Manual</u><br><br>1) Generic FReSH START Manual which includes information about self-harm, results of the FReSH START research in the first part of the programme including findings from a systematic review of what service users find helps them to stop self-harming and a Q study to understand the underlying functions of self-harm. The most common functions of self-harm described by services users are described in the manual to help guide the therapist explore client function in the initial sessions. The manual also provides guidance on risk assessment and safety planning, and the structure of therapy. Includes an appendix comprising standardised documents for risk assessment safety planning, and assessing the functions of self-harm.<br>2) ACT-specific training manual which describes: a) the principles of ACT and the rationale for application to helping people who self-harm; b) explains and exemplifies basic ACT therapeutic skills (active listening, reflection experiential methods, non-directive etc. c)) Describes and exemplifies basic ACT therapy techniques (centring, defusion, perspective-taking; d) details session plans for ACT in FRESH

		START; 5) includes the patient handouts for home practice tasks for each taught skill; e) an appendix including the ACT-fidelity measure.<br>3) ACT-specific fidelity assessment criteria rating the degree to which therapists adhered to and applied the ACT principles to their sessions will be used to assess 'trial readiness'. These criteria are rated on a 0-3 scale and focused around the previously mentioned ACT therapeutic skills and techniques.<br><br><u>Patient/Participant facing materials</u><br><br>Participants will receive: a Formulation sheet and home practice sheets outlining the skills taught in the skills practice sessions and techniques to practice between sessions, where applicable<br><br><u>Clinician training resources</u><br><br>Training resources include: 1) the aforementioned clinician manual; 2) a PowerPoint slide deck for the two-day training session; 3) Website hosting videos of role plays, manuals and all the home practice sheets.
**4.**	Procedures: Describe each of the procedures, activities, and/or processes used in the intervention, including any enabling or support activities.	There are 12 sessions (with two possible booster sessions) in FReSH START. These are divided up into four stages:<br><br><u>Sessions 1-2: Assessment</u><br><br>• Examine the function of self-harm according to the FReSH START assessment.<br>• Conduct a risk assessment.

		• Discuss and develop a safety plan if it is relevant and the participant does not have one and would like to have one.<br>• Have conversations that enable therapist and client to see: what are the clients values; what the client does when difficult thoughts and feelings are present and whether this is helpful.<br>• Home practice: Noticing task. Simply asking clients to non-reactively observe their emotions and thoughts in clinically relevant situations.<br><u>Session 3: Formulation (parts of the assessment maybe completed in this session)</u><br>• Outline a brief ‘centring task’ to bring focus to the session<br>• Undertake a task ‘Values Wheel’ to formally name values.<br>• Using the information gained in the Assessment stage and the ‘values wheel’, suggest what psychological flexibility skills participants may want to develop in the subsequent skills practice sessions to increasee life-enriching activity. Complete a standardised formulation sheet to make this more straightforward.<br>• Home practice: ‘Smallest possible step’ task: Choosing a new small (smallest possible) values-consistent activity that they can consistently do over the week.<br><u>Sessions 4 – 10: Skills Practice Sessions</u><br>• Continue with the ‘centring task, if client willing<br>• Based on the formulation, select from the 10 skills practice sections available to use in the middle 30 minute portion of each of session (Listed in the table below). These sections include exercises and reflections designed to help participants see additional ways to respond to difficult thoughts and feelings, with which they can experiment – there is a linked home practice sheet to help participants experiment between sessions.<br>• With the rest of the session, help the participant effectively to reflect on the previous week and whether any previously-learned skills seemed helpful to them in this context.

		<u>Session 11 -12: Endings</u><br><br>• Consciously orientating to finishing the work with the participant, with a focus on the relationship and reinforcing helpful actions learned over the course of therapy. The therapist aims to do two things: 1) End the relationship in a clear and compassionate way, and help them negotiate any difficulties this brings; 2) Help the participant notice the changes that they have made over your time working together, and see how they can commit to keep doing things that work.

Focus	PF Skills	Skills practice section plans	Content
Openness	Skill 1: Get unhooked from unhelpful thoughts	a. Distancing and unhooking<br>b. Letting thoughts pass	*Metaphor:* Chatty mind<br>*Exercises:* Counting; Polar Bear; "I am having the thought that"<br>*Metaphor:* Weather & Sky<br>*Exercise:* Leaves on the stream
	Skill 2. Make Space for strong emotions	a. Make space (to choose what to do next)	*Metaphor:* Emotions messengers<br>*Exercises:* Labelling emotions
Awareness	Skill 3: Be able to be in the present moment	a. Practicing being present	*Metaphor:* Chatty mind<br>*Exercises:* Centring exercises (Drop anchor; notice 3 things)
	Skill 5: Choose to be kind to yourself	a. Responding to yourself as a friend<br>b. Learning from your best relationship	*Metaphor:* Child metaphor<br>*Exercises:* See how you respond to a friend<br>*Exercise:* Your kindest other
Engagement	Skill 4. Connect with what's important	a. Connecting with what's important	*Metaphor:* Values are a compass<br>*Exercises:* Getting in touch with your values; smallest possible steps
	Apply any or all skills	a. Improving sleep quality<br>b. Improving relationship with body<br>c. Doing things other than self-harm	*Exercises:* Sleep hygiene sheet<br>*Exercises:* Building a kinder relationship body sheet<br>*Exercise:* List of things people found helpful sheet

	**WHO PROVIDED**	
**5.**	For each category of intervention provider (e.g. psychologist, nursing	

	assistant), describe their expertise, background and any specific training given.	The intervention will be delivered by liaison mental health professionals working with self-harm, most commonly nurses. Other mental health staff who work in acute mental health services may also be considered. Potential therapists will have prior expertise in the risk assessment and management of service users who present with self-harm. Potential therapists will be embedded within acute mental health services with access to a Consultant Psychiatrist or nominated deputy to help manage/discuss risk issues. There is no requirement for previous training in psychological therapy, but staff who have a prior psychological therapy training can be included. Potential therapists must have pre-existing good interpersonal skills. Potential therapists will receive online training in the FReSH START assessment and ACT for self-harm. This will consist of 6-8 on line sessions of theory, didactic teaching, small group work and role plays. All potential therapists will undergo a trial readiness assessment at the end of training to assess their basic therapeutic skills. All therapists will receive ongoing regular supervision (at least one session of supervision every two weeks) from an experienced ACT therapist whilst they are delivering treatment.
	**HOW**	
**6.**	Describe the modes of delivery (e.g. face-to-face or by some other mechanism, such as internet or telephone) of the intervention and whether it was provided individually or in a group.	The intervention is one to one and can be provided face to face or via video link or telephone.<br>If the intervention is delivered face to face it will be conducted with appropriate social distancing and appropriate degrees of personal protective equipment.<br>All sessions will be audio-recorded and encrypted using a suitable portable device.
	**WHERE**	
**7.**	Describe the type(s) of location(s) where the	Mental Health Trust clinical offices or Acute Hospital Trust offices or remote delivery.

	intervention occurred, including any necessary infrastructure or relevant features.	
	**WHEN and HOW MUCH**	
**8.**	Describe the number of times the intervention was delivered and over what period of time including the number of sessions, their schedule, and their duration, intensity or dose.	There will be 12 sessions of weekly therapy, 50 minutes- 1 hour duration. Completion of therapy is defined as either 12 sessions or a mutually agreed ending.<br><br>2 additional booster sessions are allowed at the discretion of the therapist and their supervisor.<br><br>These additional sessions will be delivered by telephone.<br><br>All sessions including booster sessions must be completed within six months of randomisation.
	**TAILORING**	
**9.**	If the intervention was planned to be personalised, titrated or adapted, then describe what, why, when, and how.	The intervention has stages and therapists must adhere to an ACT approach and use ACT techniques accordingly. However, the content of sessions is tailored to each participant, for example only skills relevant to each participants formulation should be suggested.<br><br>A dominating life problem (e.g. loss of home, end of a relationship, issue with the authorities) may mean that the focus of therapy is most appropriately focused on managing this issue. Here, a therapist will focus simply on helping the participant to notice thoughts, emotions and urges, in order to make effective choices (i.e. do things that maintain well-being and continue to enrich) in the presence of a significant stressor. In such an instance a

		therapist may decide to simply use the basic (ACT) therapy skills as the participant reviews their week, discussed the ongoing stressor and plans for the week ahead –ergo, formulation, and skills practice sections may be omitted.
	**MODIFICATIONS**	
**10.**‡	If the intervention was modified during the course of the study, describe the changes (what, why, when, and how).	During the feasibility study in preparation for this trial, the mode of therapy delivery was widened so the therapy could be delivered face to face (with appropriate social distancing), or by video conferencing or by telephone. This flexibility of delivery will be maintained for the main trial.
	**HOW WELL**	
**11.**	Planned: If intervention adherence or fidelity was assessed, describe how and by whom, and if any strategies were used to maintain or improve fidelity, describe them.	For all participants who start therapy, the initial 1-3 sessions will be rated for fidelity to safety and FReSH START components.<br><br>One additional session of each participants subsequent sessions will be rated for fidelity to therapy specific components. The session reviewed will be randomly selected from the available sessions for each participant.<br><br>O'Neill, L., Latchford, G., McCracken, L. M., & Graham, C. D. (2019). The development of the Acceptance and Commitment Therapy Fidelity Measure (ACT-FM): A delphi study and field test. *Journal of Contextual Behavioral Science*, *14*, 111-118.
**12.**‡	Actual: If intervention adherence or fidelity was	Safety components were covered in all reviewed sessions. FReSH START components were fully or partially addressed in 6 out of 7 reviewed sessions. The therapy-specific components for ACT were delivered as planned

	assessed, describe the extent to which the intervention was delivered as planned.	in 50% of reviewed sessions. The therapist training will be adapted in the definitive trial to focus on the ACT components which were not commonly addressed.

TIDieR Template for Intervention Description and Replication

Cognitive Behaviour Therapy for Self-Harm

Item number	Item	Other † (details)
	**BRIEF NAME**	
**1.**	Provide the name or a phrase that describes the intervention.	Cognitive Behaviour Therapy for Self-harm
	**WHY**	
**2.**	Describe any rationale, theory, or goal of the	**Broad theoretical underpinnings of Cognitive Behaviour Therapy**<br><br>Cognitive Behaviour Therapy (CBT) draws on behaviour theory and cognitive models of emotional distress. Cognitive models suggest that on the basis of early experiences people develop schemas, consisting of beliefs

	elements essential to the intervention.	about the self, world and the future. These can take the form of unconditional beliefs (e.g., 'I am unlovable') as well as conditional beliefs ('Unless I give in to people all of the time I will be rejected'). These beliefs are not always present; instead, they become so when a life-event meshes with the content of the schema (e.g., the experience of rejection for someone with beliefs that they are unlovable). The activation of the schema influences the way in which information is processed. This gives rise to negative thoughts, which in turn lead to emotional distress and patterns of behaviour that may maintain the thoughts and the underlying schema.<br><br>In addition to cognitive models, there are two strands of behaviour theory that underpin CBT approaches: classical and operant conditioning. Classical conditioning principles state that pairing a neutral stimulus with an unconditional stimulus (a stimulus that automatically brings about a particular response such as pain or distress) leads the neutral stimulus to become a conditional stimulus. This means that the presentation of the conditional stimulus leads to a similar response even when the unconditional stimulus is not presented. Modern learning theory accounts suggest that the conditional stimulus establishes an expectancy for the occurrence of the unconditional stimulus. Repeated presentation of the conditional stimulus in the absence of the unconditional stimulus leads to new learning: the conditional stimulus no longer signals the occurrence of the unconditional stimulus and so the response reduces. This forms the theoretical basis for exposure-based treatments that are a mainstay of CBT treatment for many presenting difficulties.<br><br>Operant conditioning principles focus on how the consequences that follow a behaviour increase or decrease the occurrence of that behaviour. Reinforcement of a behaviour occurs when a behaviour increases. Reinforcement can be positive (occurrence of a stimulus leads to increase in a behaviour) or negative (non-occurrence of a stimulus leads to an increase in behaviour). Punishment of a behaviour is defined as occurring when a behaviour reduces. Punishment can be positive (occurrence of a stimulus leads to decrease in a behaviour) or negative

		(non-occurrence of a stimulus leads to a reduction in behaviour). These principles are used in CBT to help understand how patterns of behaviour are maintained and to introduce new patterns of behaviour.<br><br>The extent to which cognitive and behavioural theories forms the basis of particular CBT protocols varies. In addition sometimes treatment techniques originally derived from behaviour theory are used with a cognitive rationale (e.g., exposure techniques are framed as a behavioural experiment to test out a particular belief).<br><br>**Application of cognitive and behavioural models to managing repeated self-harm**<br><br>CBT approaches to self-harm seek to understand the functions of the behaviour drawing broadly on operant conditioning principles while also recognising that the behaviour is triggered by cognitions that serve to facilitate the behaviour. These cognitions may relate to fundamental beliefs about the self (e.g., 'I am worthless and deserve punishment'), beliefs that facilitate the behaviour ('There is no other way to deal with how I feel at the moment') and beliefs about the consequences of the behaviour (e.g., 'This proves I am worthless', 'This proves there is no other way to cope').<br><br>Treatment consists of identify the various functions of the self-harm behaviour and the supporting cognitions. Interventions involve identifying alternative behaviours that may serve the same function, evaluating the supporting cognitions and conducting behavioural experiments to test out those cognitions.

		Cognitive and behavioural strategies may also be used to help the person with other specific difficulties they are experiencing (e.g., low mood, anxiety) or may focus more broadly in making general improvements in wellbeing (by increasing engagement in activities that are meaningful or important to the person).
	**WHAT**	
**3.**	Materials: Describe any physical or informational materials used in the intervention, including those provided to participants or used in intervention delivery or in training of intervention providers. Provide information on where the materials can be accessed (e.g. online appendix, URL).	Materials: 1) Generic FReSH START Manual which includes information about self-harm, results of the FReSH START research in the first part of the programme including findings from a systematic review of what service users find helps them to stop self-harming and a Q study to understand the underlying functions of self-harm. The most common functions of self-harm described by services users are described in the manual to help guide the therapist explore client function in the initial sessions. The manual also provides guidance on risk assessment and safety planning, and the structure of therapy.<br><br>2) CBT-specific FReSH START manual which describes the principles of CBT and the rationale for application to helping people who self-harm; explains and exemplifies basic CBT therapeutic skills (active listening, reflection) Describes and exemplifies basic CBT therapy techniques (functional analysis of self-harm behaviour and supporting cognitions around the behaviour; identifying alternative behaviours that may serve the same function as self-harm, evaluating the supporting cognitions and conducting behavioural experiments to test out those cognitions; using CBT techniques to help the person with specific difficulties they are experiencing; encouraging improvements to broad well being and maintaining CBT-fidelity<br>3) CBT-specific fidelity assessment criteria rating the degree to which therapists adhered to and applied the CBT principles to their sessions will be used to assess ‘trial readiness’. These criteria are rated on a 1-7 scale and focused around the previously mentioned CBT therapeutic skills and techniques.

		<u>Clinician training resources</u><br><br>Training resources include: 1) the aforementioned clinician manuals; 2) a PowerPoint slide deck for the two-day training session; 3) Website hosting videos of role plays, manuals and relevant client participation material.
**4.**	Procedures: Describe each of the procedures, activities, and/or processes used in the intervention, including any enabling or support activities.	There are 12 sessions (with two possible booster sessions) in FReSH START.<br><br>**Procedure for each session**<br><br>Each session uses a broadly similar structure involving a start, middle and end phase.<br><br>Start phase:<br><br>• Check in<br>• Risk assessment and safety planning<br>• Agree agenda<br><br>Middle:<br><br>• Review of between-session tasks<br>• Working through the agenda and introducing new learning

		• Agreeing new between-session tasks<br>• As necessary, revisiting the shared understanding<br>• As necessary, revisiting and revising goals<br><br>End:<br>• Summary of main points from the session<br>• Summary of the between-session task<br>• Revisit risk assessment and safety planning if needed<br>• Feedback on the session<br>• Agree time and date of the next session<br>• As necessary, discuss the number of sessions remaining.<br><br>**Structure and content of therapy**<br>This general session structure is used across the four stages of therapy, with stages one and two happening during the first few session, stage three taking up the bulk of the sessions, and stage four taking up the final two sessions. The initial 3 sessions can be used to focus on basic assessment, risk assessment and safety planning<br><br><u>Stage 1 (Sessions 1-3)</u><br>• Examine the function of self-harm according to the FReSH START assessment.<br>• Conduct a risk assessment.

		• Discuss and develop a safety plan if it is relevant and the participant does not have one and would like to have one.<br>• Finding out about the person<br>• Build the foundations of a therapeutic relationship<br>• Provide a brief introduction to the CBT approach<br>• Conduct an assessment of the person's main difficulties<br><br>Stage 2: Developing a shared understanding of current difficulties<br>• Develop a shared understanding of the self-harm based on a cognitive-behavioural formulation<br>• Develop a shared understanding of other difficulties based on a cognitive-behavioural formulation<br>• Goal setting<br><br>Stage 3: Working together to improve wellbeing<br>This may include using cognitive and behavioural techniques to:<br>• Work to manage the self-harm better<br>• Find alternative ways to achieve the same functions of the self-harm<br>• Work on other specific difficulties,<br>• A more general focus on improving wellbeing.<br><br>Stage 4: Keeping going with the changes made

		This involves the participant developing a written plan that covers:<br>• Summarising what they've learnt and what changes they've made<br>• A detailed plan to maintain the changes made<br>• Signs that difficulties may be returning<br>• Plans for what to do about major setbacks<br>• Ensuring a safety plan is in place after the end of treatment.<br><br>There is also the option of two booster telephone sessions after the end of the main phase of treatment. These follow the structure and content of the stage 4 sessions.
	**WHO PROVIDED**	
**5.**	For each category of intervention provider (e.g. psychologist, nursing assistant), describe their expertise, background and any specific training given.	The intervention will be delivered by liaison mental health professionals working with self-harm, most commonly nurses. Other mental health staff who work in acute mental health services may also be considered. Potential therapists will have prior expertise in the risk assessment and management of service users who present with self-harm. Potential therapists will be embedded within acute mental health services with access to a Consultant Psychiatrist or nominated deputy to help manage/discuss risk issues. There is no requirement for previous training in psychological therapy, but staff who have a prior psychological therapy training can be included. Potential therapists must have pre-existing good interpersonal skills. Potential therapists will receive online training in the FReSH START assessment and CBT for self-harm. This will consist of 6-8 on-line sessions of theory, didactic teaching, small group work and role plays. All potential therapists will undergo a trial readiness assessment at the end of training to assess their basic therapeutic skills. All therapists will receive ongoing

		regular supervision (at least one session of supervision every two weeks) from an experienced CBT therapist whilst they are delivering treatment.
	**HOW**	
**6.**	Describe the modes of delivery (e.g. face-to-face or by some other mechanism, such as internet or telephone) of the intervention and whether it was provided individually or in a group.	There will be 12 sessions of weekly therapy, 50 minutes- 1 hour duration. Completion of therapy is defined as either 12 sessions or a mutually agreed ending.
	**WHERE**	
**7.**	Describe the type(s) of location(s) where the intervention occurred, including any necessary infrastructure or relevant features.	Mental Health Trust clinical offices or Acute Hospital Trust offices or remote delivery.
	**WHEN and HOW MUCH**	
**8.**	Describe the number of times the intervention was delivered and over what	There will be 12 sessions of weekly therapy, 50 minutes- 1 hour duration.<br>2 additional booster sessions are allowed at the discretion of the therapist and their supervisor.

	period of time including the number of sessions, their schedule, and their duration, intensity or dose.	These additional sessions will be delivered by telephone.<br><br>All sessions including booster sessions must be completed within six months of randomisation.
	**TAILORING**	
**9.**	If the intervention was planned to be personalised, titrated or adapted, then describe what, why, when, and how.	Although there is a broad outline of session content, each session is personalised (e.g., personalised clinical formulation, personalised treatment goals, personalised behavioural experiments). This is standard practice in CBT.<br><br>A dominating life problem (e.g. loss of home, end of a relationship, issue with the authorities) may mean that the focus of therapy is most appropriately focused on managing this issue. Here, a therapist will focus simply on helping the participant to notice thoughts, emotions and urges, in order to make effective choices (i.e. do things that maintain well-being and continue to enrich) in the presence of a significant stressor. In such an instance a therapist may decide to simply use the basic (CBT) therapy skills as the participant reviews their week, discusses the ongoing stressor and plans for the week ahead.
	**MODIFICATIONS**	
**10.**[‡]	If the intervention was modified during the course of the study, describe the changes (what, why, when, and how).	During the feasibility study in preparation for this trial, the mode of therapy delivery was widened so the therapy could be delivered face to face (with appropriate social distancing), or by video conferencing or by telephone. This flexibility of delivery will be maintained for the main trial.

	**HOW WELL**	
**11.**	Planned: If intervention adherence or fidelity was assessed, describe how and by whom, and if any strategies were used to maintain or improve fidelity, describe them.	For all participants who start therapy, the initial 1-3 sessions will be rated for fidelity to safety and FReSH START components.<br><br>One additional session of each participants subsequent sessions will be rated for fidelity to therapy specific components. The session reviewed will be randomly selected from the available sessions for each participant.<br><br>Startup, M., Shapiro, D.A. Therapist treatment fidelity in prescriptive vs. exploratory psychotherapy. Br J Clin Psychol 1993; 32 (4) :443-56.
**12.**[‡]	Actual: If intervention adherence or fidelity was assessed, describe the extent to which the intervention was delivered as planned.	Safety and FReSH START components were fully addressed in 6 out of 7 reviewed sessions. The therapy-specific components for CBT were delivered as planned in 2 out of 5 reviewed sessions. The therapist training will be adapted in the definitive trial to focus on the CBT components which were not commonly addressed.
