# Supplementary material for: Results of a feasibility study of the FReSH START intervention to improve quality of life and other outcomes in people who repeatedly self-harm (Function REplacement in repeated Self-Harm: Standardising Therapeutic Assessment and the Related Therapy)
Source: Pilot Feasibility Stud. 2025 May 15;11:67. doi: 10.1186/s40814-025-01644-2 (PMC12080260; doi:10.1186/s40814-025-01644-2)
Supplement: Supplementary file 2 — Additional file 2. Details of item-level imputation of patient-reported outcome measures [file 40814_2025_1644_MOESM2_ESM.pdf]

Additional file 2. Details of item-level imputation of patient-reported outcome measures

Clinical Outcomes in Routine Evaluation - Outcome Measure (CORE-OM):

The CORE-OM is a 34-item generic measure of psychological distress scored from 0 to 4 (Barkham 2005). The overall score will be calculated as the mean score of the items multiplied by 10, giving a score from 0 to 40 with a higher score indicating higher levels of distress. Where there is missing data for 3 items or fewer, the overall score will be calculated as the mean across the recorded items multiplied by 10 (https://www.coresystemtrust.org.uk/instruments/core-om-information/). If more than 3 items are missing, the overall score will be assigned as missing.

Beck Hopelessness Scale (BHS):

The Beck Hopelessness Scale (BHS) is a questionnaire in which a patient answers "true" or "false" to a series of 20 statements that test his or her feelings about the future (Beck 1974). After inversion of the positively worded items, a sum-score is calculated between 0 and 20 with a higher score indicating higher levels of hopelessness. In the absence of official instructions for handling missing item data, we will use the half rule (Fairclough 2010). If at least half the questions in the measure (10 or more items) are answered, missing item scores will be substituted by the mean of the answered questions for that specific scale. If the half rule calculation results in a fractional value then it will be rounded to the nearest whole number. If more than 50% of the items for the specific scale / subscale are missing, the outcome will be assigned as missing.

Patient Health Questionnaire- 9 (PHQ-9):

The PHQ-9 is a nine-item measure of depressive symptoms (Kroenke 2001). Items are rated from 0 (not at all), 1 (several days), 2 (more than half the days), and 3 (nearly every day). The total score is the sum of all items and ranges from 0 to 27, with a higher score indicating higher levels of depression.

In the absence of official instructions for handling missing item data, we follow the approach defined within the NHS data dictionary: if one or two items are missing, then they will be substituted with the average score of the non-missing items when calculating the total score (https://www.datadictionary.nhs.uk/data_dictionary/nhs_business_definitions/p/patient_health_questionnaire-9_de.asp). If there are more than two missing items, the total score will be assigned as missing.

Social Connectedness Scale – revised (SCS-R):

The Social Connectedness Scale – Revised is a 20-item measure (Lee 2001, Lee 1995). Items that are negatively-worded will be re-coded to range from 1 ‘strongly agree’ to 6 ‘strongly disagree’. The overall score will calculated as the total of the 20 items, ranging from 20 to 120. A higher score indicates more connectedness to others.

In the absence of official instructions for handling missing item data, we will use the half rule (Fairclough 2010). If at least half the questions in the measure (10 or more items) are answered, missing item scores will be substituted by the mean of the answered questions for that specific scale. If the half rule calculation results in a fractional value then it will be rounded to the nearest whole number. If more than 50% of the items for the specific scale / subscale are missing, the outcome will be assigned as missing.

References:

Barkham 2005: Barkham M, Gilbert N, Connell J, Marshall C, Twigg E. Suitability and utility of the CORE-OM and CORE-A for assessing severity of presenting problems in psychological therapy services based in primary and secondary care settings. Br J Psychiatry. 2005;186:239-46.

Beck 1974: Beck AT, Weissman A, Lester D, Trexler L. The measurement of pessimism: the hopelessness scale. J Consult Clin Psychol. 1974;42(6):861-5.

Fairclough 2010: Fairclough DL. Design and analysis of quality of life studies in clinical trials: Chapman and Hall/CRC; 2010.

Kroenke 2001: Kroenke K, Spitzer RL, Williams JB. The PHQ-9: validity of a brief depression severity measure. Journal of general internal medicine. 2001;16(9):606-13

Lee 1995: Lee RM, Robbins SB. Measuring belongingness: The Social Connectedness and the Social Assurance scales. Journal of Counseling Psychology. 1995;42(2):232-41.

Lee 2001: Lee RM, Draper M, Lee S. Social connectedness, dysfunctional interpersonal behaviors, and psychological distress: Testing a mediator model. Journal of Counseling Psychology. 2001;48(3):310-8.

https://www.datadictionary.nhs.uk/data_dictionary/nhs_business_definitions/p/patient_health_questionnaire-9_de.asp

https://www.coresystemtrust.org.uk/instruments/core-om-information/
