# Supplementary material for: Results of a feasibility study of the FReSH START intervention to improve quality of life and other outcomes in people who repeatedly self-harm (Function REplacement in repeated Self-Harm: Standardising Therapeutic Assessment and the Related Therapy)
Source: Pilot Feasibility Stud. 2025 May 15;11:67. doi: 10.1186/s40814-025-01644-2 (PMC12080260; doi:10.1186/s40814-025-01644-2)
Supplement: Supplementary file 3 — Additional file 3. Baseline characteristics comparing those who were followed-up or not [file 40814_2025_1644_MOESM3_ESM.pdf]

Additional file 3. Baseline characteristics comparing those who were followed-up or not

	Followed-up	Not followed-up	Overall
**Age (years)**			
Mean (s.d.)	25.1 (10.04)	25.4 (8.37)	25.2 (9.14)
Median (range)	22 (18, 58)	23 (19, 46)	23 (18, 58)
Missing	0	0	0
N	16	14	30
**Gender**			
Male	3 (18.8%)	2 (14.3%)	5 (16.7%)
Female	12 (75.0%)	11 (78.6%)	23 (76.7%)
Non-binary	0 (0.0%)	1 (7.1%)	1 (3.3%)
Prefer not to say	1 (6.3%)	0 (0.0%)	1 (3.3%)
Other	0 (0.0%)	0 (0.0%)	0 (0.0%)
**Type of self-harm for index event**			
Self-injury	6 (37.5%)	6 (42.9%)	12 (40.0%)
Self-poisoning	6 (37.5%)	5 (35.7%)	11 (36.7%)
Both	4 (25.0%)	3 (21.4%)	7 (23.3%)
**CORE-OM Total score**			
Mean (s.d.)	20.2 (5.92)	21.9 (5.58)	21.0 (5.73)
Median (range)	18 (12, 32)	22 (14, 34)	21 (12, 34)
Missing	0	0	0
N	16	14	30
**BHS Total score**			
Mean (s.d.)	13.3 (4.70)	15.6 (4.78)	14.4 (4.80)
Median (range)	13 (4, 20)	17 (3, 20)	15 (3, 20)
Missing	0	0	0
N	16	14	30

	Followed-up	Not followed-up	Overall
**PHQ-9 Total score**			
Mean (s.d.)	18.6 (5.49)	18.9 (5.11)	18.7 (5.23)
Median (range)	19 (9, 26)	17 (14, 26)	19 (9, 26)
Missing	0	0	0
N	16	14	30
**SCS-R Total score**			
Mean (s.d.)	59.0 (15.45)	59.4 (20.54)	59.2 (17.68)
Median (range)	56 (35, 90)	57 (22, 101)	57 (22, 101)
Missing	0	0	0
N	16	14	30
