# Supplementary material for: Results of a feasibility study of the FReSH START intervention to improve quality of life and other outcomes in people who repeatedly self-harm (Function REplacement in repeated Self-Harm: Standardising Therapeutic Assessment and the Related Therapy)
Source: Pilot Feasibility Stud. 2025 May 15;11:67. doi: 10.1186/s40814-025-01644-2 (PMC12080260; doi:10.1186/s40814-025-01644-2)
Supplement: Supplementary file 4 — Additional file 4. Text message response rate by whether returned questionnaire or not [file 40814_2025_1644_MOESM4_ESM.pdf]

Additional file 4. Text message response rate by whether returned questionnaire or not

Additional file 4.1 Text message response rate by whether returned questionnaire or not: SMS question on general health

	Followed-up	Not followed-up	Overall
**Response to SMS question on general health (yes/no) - month 1**			
Responded	15 (93.8%)	8 (72.7%)	23 (85.2%)
Did not respond	1 (6.3%)	3 (27.3%)	4 (14.8%)
**Response to SMS question on general health (yes/no) - month 2**			
Responded	11 (68.8%)	6 (66.7%)	17 (68.0%)
Did not respond	5 (31.3%)	3 (33.3%)	8 (32.0%)
**Response to SMS question on general health (yes/no) - month 3**			
Responded	14 (87.5%)	4 (44.4%)	18 (72.0%)
Did not respond	2 (12.5%)	5 (55.6%)	7 (28.0%)
**Response to SMS question on general health (yes/no) - month 4**			
Responded	10 (62.5%)	4 (44.4%)	14 (56.0%)
Did not respond	6 (37.5%)	5 (55.6%)	11 (44.0%)
**Response to SMS question on general health (yes/no) - month 5**			
Responded	10 (62.5%)	5 (55.6%)	15 (60.0%)
Did not respond	6 (37.5%)	4 (44.4%)	10 (40.0%)
**Response to SMS question on general health (yes/no) - month 6**			
Responded	11 (68.8%)	2 (22.2%)	13 (52.0%)
Did not respond	5 (31.3%)	7 (77.8%)	12 (48.0%)

Additional file 4.2 Text message response rate by whether returned questionnaire or not: SMS question on self-harm

	Followed-up	Not followed-up	Overall
**Response to SMS question on self-harm (yes/no) - month 1**			
Responded	15 (93.8%)	7 (63.6%)	22 (81.5%)
Did not respond	1 (6.3%)	4 (36.4%)	5 (18.5%)
Total	16 (100%)	11 (100%)	27 (100%)
**Response to SMS question on self-harm (yes/no) - month 2**			
Responded	12 (75.0%)	7 (77.8%)	19 (76.0%)
Did not respond	4 (25.0%)	2 (22.2%)	6 (24.0%)
Total	16 (100%)	9 (100%)	25 (100%)
**Response to SMS question on self-harm (yes/no) - month 3**			
Responded	13 (81.3%)	4 (44.4%)	17 (68.0%)
Did not respond	3 (18.8%)	5 (55.6%)	8 (32.0%)
Total	16 (100%)	9 (100%)	25 (100%)
**Response to SMS question on self-harm (yes/no) - month 4**			
Responded	13 (81.3%)	4 (44.4%)	17 (68.0%)
Did not respond	3 (18.8%)	5 (55.6%)	8 (32.0%)
Total	16 (100%)	9 (100%)	25 (100%)
**Response to SMS question on self-harm (yes/no) - month 5**			
Responded	11 (68.8%)	5 (55.6%)	16 (64.0%)
Did not respond	5 (31.3%)	4 (44.4%)	9 (36.0%)
Total	16 (100%)	9 (100%)	25 (100%)
**Response to SMS question on self-harm (yes/no) - month 6**			
Responded	10 (62.5%)	3 (33.3%)	13 (52.0%)
Did not respond	6 (37.5%)	6 (66.7%)	12 (48.0%)
Total	16 (100%)	9 (100%)	25 (100%)
