# Supplementary material for: Results of a feasibility study of the FReSH START intervention to improve quality of life and other outcomes in people who repeatedly self-harm (Function REplacement in repeated Self-Harm: Standardising Therapeutic Assessment and the Related Therapy)
Source: Pilot Feasibility Stud. 2025 May 15;11:67. doi: 10.1186/s40814-025-01644-2 (PMC12080260; doi:10.1186/s40814-025-01644-2)
Supplement: Supplementary file 5 — Additional file 5. Health Economics Costs Analysis [file 40814_2025_1644_MOESM5_ESM.pdf]

Additional file 5: Health Economics Costs Analysis

Intervention Costs

Intervention costs were calculated as fixed costs (which are the same for all participants) for training and supervision, and variable costs (which differ by participants) for intervention delivery and administrative tasks. Costs are reported separately by treatment, and were calculated by multiplying the time required for therapists and supervisors by their associated unit costs. Therapists were assumed to be mental health nurses at AFC band 6, whilst supervisors were assumed to be AFC band 7.

In the base case analysis, training costs were not included as a direct intervention costs but viewed similarly to general qualifications i.e. as an investment in the healthcare professionals' human capital over their remaining career. Therefore, training was incorporated into the intervention costs by adjusting unit costs, following methods used by NICE (2022): The mean cost of training per therapist was calculated based on recorded training durations in the feasibility study case report forms (Table A5.1). This cost was annuitised and added to unit cost calculations using a formula by Netten (1998) and assuming 42 years up to retirement, 23 years of useful working life remaining and an annual discount rate of 3.5% (Table A5.2). The adjusted unit costs were applied to all therapist time required for intervention delivery and supervision. A sensitivity analysis was also conducted, where training costs were included directly within the intervention cost calculation, by summing the total cost of training across all therapists and distributing this fixed cost equally across participants (Table A5.2).

Table A5.1: Training Costs (£ GBP, 2021)

	Total	ACT	CBT	PIT
Therapists (n)	28	8	10	10
Training Costs 1				
Training duration (hours)	492.00	180.00	102.00	210.00
Training cost for all therapists	£29,756	£10,886	£6,169	£12,701
Mean training costs per therapist	£1,063	£1,361	£617	£1,270
Annutised training cost per therapist 2	£91.95	£117.73	£53.37	£109.86

1: Training costs calculated assuming unit cost per hour of £60.48 for Nurse AFC band 6
2: Annutised costs calculated using formula by Netten et al., (1998), assuming 42 years up to retirement, 23 years of useful working life remaining, and an annual discount rate of 3.5%.

Table A5.2: Unit Costs for Therapists and Supervisors (£ GBP, 2021)

	Therapists: AFC Band 6				Supervisors: AFC Band 7
	Unadjusted	Adjusted			
		ACT	CBT	PIT	
Wages/ Salary	£35,118	£35,118	£35,118	£35,118	£42,376
Salary oncosts	£10,889	10,889	£10,889	10,889	£13,391
Qualifications					
General qualifications	£8,744	£8,744	£8,744	£8,744	£8,744
Training Costs 1	N/A	£118	£53	£110	N/A
Overheads					
Management, admin, estates staff	£14,396	£14,396	£14,396	£14,396	£17,450
Non-staff	£20,887	£20,887	£20,887	£20,887	£25,318
Capital overheads	£5,102	£5,102	£5,102	£5,102	£5,102
Working time (hours)	1,573	1,573	1,573	1,573	1,573
Unit cost per hour	£60.48	£60.56	£60.51	£60.55	£71.44

Notes: Cost table obtained from PSSRU; Therapists' adjusted unit costs are used for the base case analysis and for scenario 2. Therapists' unadjusted unit costs are used in scenario 1.
1: Annutised training costs obtained from Table A5.1.

Supervision costs (Table A5.3) were the time required for supervisors and therapists during supervision sessions, both of which were recorded in case report forms. Per participant costs were calculated by distributing the total supervision costs equally across participants. Supervision costs in the feasibility study may be higher than expected in the definitive trial due to a relatively high number of therapists (n=28) and low number of participants recruited (n=30). Therefore a sensitivity

analysis was conducted (scenario 2), where the therapists costs for attending supervision sessions were distributed per participant, based on assumed ratio of 1 therapist per 5 participants that may be more representative of the definitive trial.

Table A5.3: Supervision costs (£ GBP, 2021)

	Total	ACT	CBT	PIT
Supervisors				
Total duration (hours)	91.67	25.58	37.92	28.17
Total cost 1	£6,549	£1,827	£2,709	£2,012
Therapists				
Total duration (hours)	156.17	28.25	67.42	60.50
Total cost 2	£9,454	£1,711	£4,080	£3,663

1: Unit costs applied for AFC band 7 as reported in Table A5.2.
2: Adjusted unit costs applied per treatment allocation as reported in Table A5.2.

Variable costs were the time required for therapists to deliver sessions and for administrative duties. Each session duration was recorded directly in case report forms, with an additional 5 minutes of time included for each missed or cancelled session. It was assumed that therapists would require 5 minutes of time on administrative tasks for every 60 minutes spent delivering therapy. Variable costs are reported in Table A5.4.

Table A5.4: Variable Costs (£ GBP, 2021): Mean (SD)

	Total (n=30)	ACT (n=8)	CBT (n=11)	PIT (n=11)
Therapy sessions duration (hours)	6.42 (4.91)	8.29 (4.44)	4.75 (5.23)	6.72 (4.77)
Therapy session costs 1	£389 (£297)	£502 (£269)	£287 (£317)	£407 (£289)
Administration (duration hours)	0.54 (0.41)	0.69 (0.37)	0.40 (0.44)	0.56 (0.40)
Administration costs 1	£32 (£25)	£42 (£22)	£24 (£26)	£34 (£24)

1: Costs for the base case applied adjusted unit costs as reported in Table A5.2.

Total intervention costs per participant are summarised in Table A5.5. In the base case analysis total costs for ACT, CBT and PIT were £986, £929 and £957 respectively. Intervention costs varied

substantially according to the costing assumptions applied for both training (scenario 1) and supervision (scenario 2).

Table A5.5: Intervention costs per participant, (£ GBP, 2021)

	**Total (n=30)**	**ACT (n=8)**	**CBT (n=11)**	**PIT (n=11)**
**Base case**				
Fixed Costs				
Training	N/A	N/A	N/A	N/A
Supervision	£533	£442	£617	£516
Variable Costs				
Therapy	£389	£502	£287	£407
Administration	£32	£42	£24	£34
Total Costs	*£955*	*£986*	*£929*	*£957*
**Scenario 1**				
Fixed Costs				
Training	£992	£1,361	£561	£1,155
Supervision	£533	£442	£617	£516
Variable Costs				
Therapy	£388	£501	£287	£406
Administration	£32	£42	£24	£34
Total Costs	*£1,946*	*£2,346*	*£1,489*	*£2,111*
**Scenario 2**				
Fixed Costs				
Training	N/A	N/A	N/A	N/A
Supervision	£286	£271	£328	£256
Variable Costs				
Therapy	£389	£502	£287	£407
Administration	£32	£42	£24	£34
Total Costs	*£707*	*£815*	*£639*	*£697*

Notes: Base case analysis incorporates training costs through adjusted therapist unit costs; Scenario 1 include training costs directly; scenario 2 includes training as per the base case analysis and adjusts supervision costs for therapists by assuming a ratio of 1 therapist to 5 participants.

Service utilisation

Participants completed a healthcare resource utilisation questionnaire, which included questions on hospital outpatient attendance (yes/no), community care appointments (number of practice visits and telephone appointments), and current prescriptions for mental health related conditions (drug name, dose and duration). Community care and outpatient resource usage was obtained at baseline and follow up, whilst medication usage was only available at the follow up. The questionnaires asked

participants to report resource usage over the previous 3-months, therefore costs for the feasibility study are not obtained across the full 6-month follow up period. In the definitive trial costs will be collated over the full follow up period as questionnaires will be administered every 3-months. Unit costs were obtained from NHS reference costs for outpatient appointments (Table 5) (NHS), the Personal Social Services Research Unit (Unit Costs of Health and Social Care 2020/21 edition for community care (Table A5.6) (PSSRU), and the British National Formulary (BNF) for medication (Table A5.7) (BNF).

Table A5.6: Healthcare unit costs, (£ GBP, 2021)

Healthcare professional	Unit Cost (per hour)	Duration (hours)		Cost		Source
		Visit	Call [1]	Visit	Call	
GP	£255	0.15	0.13	£38.25	£33.15	PSSRU
Mental health worker	£57	0.25	0.13	£14.25	£7.41	PSSRU
Help-line	£44	0.25	0.13	£11.00	£5.72	PSSRU
Other	£44	0.25	0.13	£11.00	£5.72	PSSRU
Drug & alcohol worker	£57	0.25	0.13	£14.25	£7.41	PSSRU
Practice nurse	£44	0.25	0.13	£11.00	£5.72	PSSRU
District nurse	£55	0.25	0.13	£13.75	£7.15	PSSRU
Social Worker	£52	0.38	0.13	£19.76	£6.76	PSSRU
Occupational therapist	£50	0.25	0.13	£12.50	£6.50	PSSRU
Hospital outpatient [2]	£202	NA	NA	NA	NA	NHS

1: Mean call duration obtained from Edwards et al. (2017).

2: Outpatient costs calculated as weighted average across all emergency and mental health related NHS Reference cost outpatient codes: 180 Accident & Emergency; 656 Clinical Psychology; 710 Adult Mental Illness; 713 Psychotherapy; 720 Eating Disorders; 721 Addiction Services; 722 Liaison Psychiatry; 725 Mental Health Recovery and Rehabilitation Services.

Table A5.7: Medication costs, (£ GBP, 2021)

Drug Name	Daily dose	Cost pack	Pack size (n doses)	Cost per day	Source
Citalopram	20mg	£0.85	28	£0.03	BNF
Mirtazapine	45mg	£1.46	28	£0.05	BNF
Sertraline	100mg	£1.37	28	£0.05	BNF

Aripiprazole	15mg	£1.18	28	£0.04	BNF
Fluoxetine	20mg	£0.83	30	£0.03	BNF
Escitalopram	10mg	£1.08	28	£0.04	BNF
Venlafaxine	75mg	£2.45	56	£0.04	BNF

Wider societal costs included work absenteeism and participants' out of pocket expenditure and were obtained at baseline and 6-months. Costs for absenteeism were calculated using the human capital method by multiplying gross wages by work days missed due to health problems. The number of work days missed was obtained from participant questionnaires, and were similarly reported over the previous 3-months and not the full 6-month follow up period. Gross wages were obtained using Office for National Statistics (ONS) mean estimates based on participants' self-reported employment status, age, and gender (ONS). Out of pocket expenditure included any items related to self-harm (e.g. for treatment or self-help material) and was reported directly by participants in the questionnaires as £ GBP.

Outpatient, community care, medication and societal costs are reported in full in Table A5.8.

Table A5.8: Outpatient, community care, medication, and societal resource usage and costs (£ GBP, 2021)

Characteristic	Baseline (all participants, n=30)		Baseline (if followed up, n=16)		Follow-up (n=16)	
	Costs	Res. Use	Costs	Res. Use	Costs	Res. Use
Outpatient						
Mean (SD)	£47.23 (£87.07)	0.23 (0.43)	£50.60 (£90.52)	0.25	£25.30 (£69.14)	0.13 (0.34)
Median	£0.00	0.00	£0.00	0.00	£0.00	0.00
Range	£0.00, £202.41	0.00, 1.00	£0.00, £202.41	0.00, 1.00	£0.00, £202.41	0.00, 1.00
95% CI	£14.72, £79.74	0.07, 0.39	£2.37, £98.84	0.01, 0.49	£11.54, £62.14	0.01, 0.25
(Missing)	0	0	0	0	0	0
Community Care						
Total Community Care						
Mean (SD)	£229.54 (£200.82)	NA	£244.05 (£226.39)	NA	£207.65 (£170.86)	NA
Median	£161.20	NA	£182.32	NA	£182.32	NA
Range	£22.23, £924.08	NA, NA	£33.15, £536.44	NA, NA	£0.00, £455.17	NA, NA
95% CI	£153.16, 305.93	NA, NA	£123.42, £364.69	NA, NA	£116.61, 298.70	NA, NA
(Missing)	1	NA	0	NA	0	NA
GP						
Mean (SD)	£162.84 (£148.20)	4.86 (4.42)	£184.77 (153.06)	5.56 (4.59	£151.62 (£130.10)	4.56 (3.93)
Median	£132.60	4.00	£149.18	4.50	£116.02	3.50
Range	£0.00, £536.44	0.00, 16.00	£33.15, £536.44	1.00, 16.00	£0.00, £397.80	0.00, 12.00
95% CI	£106.47, £219.22	3.18, 6.54	£103.22, £266.33	3.12, 8.01	£82.30, £220.95	14
(Missing)	1	1	0	0	0	0

Characteristic	Baseline (all participants, n=30)		Baseline (if followed up, n=16)		Follow-up (n=16)	
	Costs	Res. Use	Costs	Res. Use	Costs	Res. Use
Mental Health Worker						
Mean (SD)	£25.45 (£56.88)	2.45 (5.39)	£14.93 (£43.81)	1.44 (4.27)	£29.43 (£57.95)	2.88 (5.32)
Median	£0.00	0.00	£0.00	0.00	£0.00	0.00
Range	£0.00, £231.42	0.00, 22.00	£0.00, £173.85	0.00, 17.00	£0.00, £174.42	0.00, 18.00
95% CI	£3.82, £47.09	0.40, 4.50	£0.00, £38.27	0.00, 3.71	£0.00, £60.30	0.04, 5.71
(Missing)	1	1	0	0	0	0
Helpline						
Mean (SD)	£14.99 (£40.08)	2.62 (7.01)	£7.51 (£13.00)	1.31 (2.27)	£2.86 (£5.91)	0.50 (1.03)
Median	£0.00	0.00	£0.00	0.00	£0.00	0.00
Range	£0.00, £205.92	0.00, 36.00	£0.00, £45.76	0.00, 8.00	£0.00, £17.16	0.00, 3.00
95% CI	£0.00, £30.24	0.00, 5.29	£0.75, £14.43	0.10, 2.52	£0.00, £6.01	0.00, 1.05
(Missing)	1	1	0	0	0	0
Drug and Alcohol Worker						
Mean (SD)	£1.53 (£8.26)	0.21 (1.11)	£2.78 (£11.12)	0.38 (1.50)	£6.91 (£20.03)	0.88 (2.50)
Median	£0.00	0.00	£0.00	0.00	£0.00	0.00
Range	£0.00, £44.46	0.00, 6.00	£0.00, £44.46	0.00, 6.00	£0.00, £73.53	0.00, 9.00
95% CI	£0.00, £4.67	0.00, 0.63	£0.00, £8.70	0.00, 1.17	£0.00, £17.58	0.00, 2.21
(Missing)	1	1	0	0	0	0
Practice Nurse						
Mean (SD)	£9.68 (£24.14)	0.90 (2.19)	£7.56 (£17.84)	0.69 (1.62)	£9.02 (£12.82)	1.00 (1.51)
Median	£0.00	0.00	£0.00	0.00	£0.00	0.00

Characteristic	Baseline (all participants, n=30)		Baseline (if followed up, n=16)		Follow-up (n=16)	
	Costs	Res. Use	Costs	Res. Use	Costs	Res. Use
Range	£0.00, £110.00	0.00, 10.00	£0.00, £66.00	0.00, 6.00	£0.00, £39.16	0.00, 5.00
95% CI	£0.56, £18.86	0.06, 1.73	£0.00, £17.07	0.00, 1.55	£2.19, £15.85	0.20, 1.80
(Missing)	1	1	0	0	0	0
Other (community care)						
Mean (SD)	£14.35 (£70.78)	2.00 (9.64)	£25.66 (£95.11)	3.56 (12.94)	£5.20 (£14.24)	0.56 (1.55)
Median	£0.00	0.00	£0.00	0.00	£0.00	0.00
Range	£0.00, £391.92	0.00, 52.00	£0.00, £381.92	0.00, 52.00	£0.00, £55.44	0.00, 6.00
95% CI	£0.00, £41.28	0.00, 5.67	£0.00, £76.34	0.00, 10.46	£0.00, £12.79	0.00, 1.39
(Missing)	1	1	0	0	0	0
Social Worker						
Mean (SD)	£0.47 (£2.51)	0.07 (0.37)	£0.85 (£3.38)	0.13 (0.50)	£0.00 (£0.00)	0.00 (0.00)
Median	£0.00	0.00	£0.00	0.00	£0.00	0.00
Range	£0.00, £13.52	0.00, 2.00	£0.00, £13.52	0.00, 2.00	£0.00, £0.00	0.00, 0.00
95% CI	£0.00, £1.42	0.00, 0.21	£0.00, £2.65	0.00, 0.39	NA, NA	NA, NA
(Missing)	1	1	0	0	0	0
District Nurse						
Mean (SD)	£0.00 (£0.00)	0.00 (0.00)	£0.00 (£0.00)	0.00 (0.00)	£2.61 (£10.45)	0.25 (1.00)
Median	£0.00	0.00	£0.00	0.00	£0.00	0.00
Range	£0.00, £0.00	0.00, 0.00	£0.00, £0.00	0.00, 0.00	£0.00, £41.80	0.00, 4.00
95% CI	NA, NA	NA, NA	NA, NA	NA, NA	£0.00, £8.18	0.00, 0.78
(Missing)	1	1	0	0	0	0

**Characteristic**	**Baseline (all participants, n=30)**		**Baseline (if followed up, n=16)**		**Follow-up (n=16)**	
	**Costs**	**Res. Use**	**Costs**	**Res. Use**	**Costs**	**Res. Use**
Occupational Therapist						
Mean (SD)	£0.22 (£1.21)	0.03 (0.19)	£0.00 (£0.00	0.00 (0.00)	£0.00 (£0.00)	0.00 (0.00)
Median	£0.00	0.00	£0.00	0.00	£0.00	0.00
Range	£0.00, £6.50	0.00, 1.00	£0.00, £0.00	0.00, 0.00	£0.00, £0.00	0.00, 0.00
95% CI	£0.00, £0.68	0.00, 0.11	NA, NA	NA, NA	NA, NA	NA, NA
(Missing)	1	1	0	0	0	0
Residential Stay						
Mean (SD)	£0.00 (£0.00)	0.00 (0.00)	£0.00 (£0.00)	0.00 (0.00)	£0.00 (£0.00)	0.00 (0.00)
Median	£0.00	0.00	£0.00	0.00	£0.00	0.00
Range	£0.00, £0.00	0.00, 0.00	£0.00, £0.00	0.00, 0.00	£0.00, £0.00	0.00, 0.00
95% CI	NA, NA	NA, NA	NA, NA	NA, NA	NA, NA	NA, NA
(Missing)	1	1	0	0	2	1
Medication Costs						
Mean (SD)	NA	NA	NA	NA	£3.44 (£3.61)	NA
Median	NA	NA	NA	NA	£2.34	NA
Range	NA, NA	NA, NA	NA, NA	NA, NA	£0.00, £8.92	NA, NA
95% CI	NA, NA	NA, NA	NA, NA	NA, NA	£1.44, £5.44	NA, NA
(Missing)	NA	NA	NA	NA	1	NA
Societal Costs						

**Characteristic**	**Baseline (all participants, n=30)**		**Baseline (if followed up, n=16)**		**Follow-up (n=16)**	
	**Costs**	**Res. Use**	**Costs**	**Res. Use**	**Costs**	**Res. Use**
Out of Pocket Expenses						
Mean (SD)	£34.38 (£60.36)	NA	£48.56 (£73.67)	NA	£35.11 (£78.44)	NA
Median	£8.50	NA	£9.50	NA	£1.10	NA
Range	£0.00, £200.00	NA, NA	£0.00, £200.00	NA, NA	£0.00, £295.00	NA, NA
95% CI	£11.84, £56.92	NA, NA	£9.30, £87.82	NA, NA	£0.00, £76.90	NA, NA
(Missing)	0	NA	0	NA	0	NA
Absenteeism						
Mean (SD)	£77.79 (£180.29)	NA	£101.07 (£229.61)	NA	£23.33 (£55.89)	NA
Median	£0.00	NA	£0.00	NA	£0.00	NA
Range	£0.00, £868.51	NA, NA	£0.00, £868.51	NA, NA	£0.00, £180.95	NA, NA
95% CI	£10.46, £145.11	NA, NA	£0.00, £223.42	NA, NA	£0.00, £55.59	NA, NA
(Missing)	0	NA	0	NA	2	NA

References:

NICE (2022): National Institute for Health and Care Excellence (NICE) (2022). "Self-harm: assessment, management and preventing recurrence. NICE guideline [NG225]. Evidence Review [J] Evidence reviews for psychological and psychosocial interventions". https://www.nice.org.uk/guidance/ng225/evidence

Netten (1998): Netten, A., Knight, J., Dennett, J., Cooley, R. & Slight, A. (1998) Development of a ready reckoner for staff costs in the NHS, Vols 1 & 2, Personal Social Services Research Unit, University of Kent, Canterbury.

PSSRU: Curtis, L. & Burns, A. (2020) Unit Costs of Health and Social Care 2020, Personal Social Services Research Unit, University of Kent, Canterbury.

NHS: NHS England. 2020/21 National Cost Collection data. National schedule of NHS costs. [cited 2022 Aug 24]. Available from: https://www.england.nhs.uk/costing-in-the-nhs/national-cost-collection/

BNF: Joint Formulary Committee. British National Formulary (online) London: BMJ Group and Pharmaceutical Press [updated 2022 July 27; cited 2022 Aug 24]. Available from: https://bnf.nice.org.uk/

Edwards et al. (2017): Edwards, H.B., Marques, E., Hollingworth, W., Horwood, J., Farr, M., Bernard, E., Salisbury, C. and Northstone, K., 2017. Use of a primary care online consultation system, by whom, when and why: evaluation of a pilot observational study in 36 general practices in South West England. BMJ open, 7(11), p.e016901

ONS: ONS (2021) "Earnings and hours worked, age group: ASHE Table 6". Office for National Statistics (ONS). [cited 2022 Aug 24] Available from: https://www.ons.gov.uk/employmentandlabourmarket/peopleinwork/earningsandworkinghours/datasets/agegroupashetable6
